# Cost‐effectiveness of a policy‐based intervention to reduce melanoma and other skin cancers associated with indoor tanning*

**DOI:** 10.1111/bjd.21046

**Published:** 2022-05-18

**Authors:** Martin Eden, Rob Hainsworth, Louisa G. Gordon, Tracy Epton, Paul Lorigan, Lesley E. Rhodes, Richard Marais, Adele C. Green, Katherine Payne

**Affiliations:** ^1^ Manchester Centre for Health Economics, Division of Population Health, Health Services Research and Primary Care, School of Health Sciences The University of Manchester Manchester UK; ^2^ Population Health Department QIMR Berghofer Medical Research Institute Brisbane Australia; ^3^ School of Nursing Queensland University of Technology (QUT) Brisbane Australia; ^4^ School of Public Health University of Queensland Brisbane Australia; ^5^ Manchester Centre for Health Psychology, Division of Psychology & Mental Health, School of Health Sciences The University of Manchester Manchester UK; ^6^ The Christie NHS Foundation Trust Manchester UK; ^7^ Division of Cancer Sciences, School of Medical Sciences The University of Manchester Manchester UK; ^8^ Centre for Dermatology Research, Division of Musculoskeletal and Dermatological Sciences, School of Biological Sciences, Faculty of Biology Medicine and Health The University of Manchester and Salford Royal NHS Foundation Trust, Manchester Academic Health Science Centre Manchester UK; ^9^ Cancer Research UK Manchester Institute The University of Manchester Manchester UK

## Abstract

**Background:**

The use of indoor tanning devices causes melanoma and other skin cancers with resulting morbidity, mortality and increased healthcare costs. Policymakers require robust economic evidence to inform decisions about a possible ban of such devices to mitigate these burdens.

**Objectives:**

To assess the health costs and consequences of introducing a policy‐based intervention across England to ban commercial indoor tanning with an accompanying public information campaign.

**Methods:**

A cost‐effectiveness analysis, adopting a healthcare system perspective, was conducted using a decision model to track a national cohort of 18‐year‐olds over a lifetime time horizon. A nationwide ban on commercial indoor tanning combined with a public information campaign (the policy‐based intervention) was compared with the status quo of availability of commercial indoor tanning. The expected costs (currency, GBP; price year, 2019) and quality‐adjusted life‐years (QALYs) were calculated. Net monetary benefit (NMB) (net benefit measured in cost compared with an accepted threshold) and net health benefit (NHB) (net gain in QALYs compared with an accepted threshold) of implementation were calculated. A probabilistic sensitivity analysis was used to calculate the probability that the intervention was cost‐effective.

**Results:**

Compared with the current situation, a ban on commercial indoor tanning combined with a public information campaign would result in 1206 avoided cases of melanoma, 207 fewer melanoma deaths and 3987 averted cases of keratinocyte cancers over the lifetime of all 18‐year‐olds (*n* = 618 873) living in England in 2019. An additional 497 QALYs would be realized along with healthcare cost‐savings of £697 858. This intervention would result in an NMB of £10.6m and an NHB of 530 QALYS. Multiple sensitivity analyses confirmed the robustness of the findings. At a cost‐effectiveness threshold of £20 000, there is a 99% likelihood of this policy‐based intervention being cost‐effective.

**Conclusions:**

The implementation of a ban on commercial indoor tanning across England with an accompanying public information campaign would be an effective use of healthcare resources.

## Graphical abstract

**What is already known about this topic?**

- Melanoma and keratinocyte skin cancers have a significant impact on population health and healthcare budgets, and a proportion are attributable to indoor tanning.
- Policy‐based interventions that restrict or ban the commercial provision of indoor tanning are in place around the world to reduce the burden of skin cancer, but economic evidence to support decision making is lacking.

**What does this study add?**

- Banning indoor tanning, supported by a public health campaign, would be an efficient use of healthcare resources to reduce melanoma and keratinocyte cancers in England.
- This policy‐intervention would save lives (4.6% melanoma deaths avoided) and reduce skin cancer treatment costs with a high degree of confidence.
- The structured and transparent analysis means that decision makers in other jurisdictions can assess the relevance of these findings to their context.

The use of indoor tanning devices causes harm to individuals and increases healthcare costs. A model‐based cost‐effectiveness anlaysis was used to estimate the economic impact of a policy‐based intervention to reduce skin cancers associated with indoor tanning. Implementation of the intervention across England would realise health gains and reduce healthcare costs.

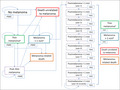

**Linked Comment:** M. Janda and C. Sinclair. Br J Dermatol 2022; 187:7.

Plain language summary available online

The use of indoor tanning devices for nonmedical purposes harms both the skin and the eyes. In 2009, the World Health Organization classified indoor tanning devices (also known as, and hereafter termed, ‘sunbeds’) as carcinogenic.^1^ Despite some evidence of decreasing use of commercial sunbeds, the practice of indoor tanning is still widespread in many countries.^2^

People who have used a sunbed increase their risk of melanoma by almost 60%.^3^ Incidence rates of basal cell carcinoma (BCC) and squamous cell carcinoma (SCC), collectively known as keratinocyte cancers (KCs), are also increased through sunbed use^4^ and, although less commonly life‐threatening, KCs are far more prevalent than melanoma. The high cost of diagnosing and treating melanoma and KC among users of sunbeds places financial burdens on healthcare systems of countries where sunbeds are popular.

Various strategies to reduce the harms associated with indoor tanning include increased taxation, public health campaigns and regulations restricting availability of commercial sunbeds.^5^ Outright bans of commercial sunbeds have been introduced in Brazil, Australia and Iran^6^ and there have been increasing calls from dermatological and oncological organizations to other jurisdictions, including populations that fall under the remit of the National Health Service (NHS) in England, to ban sunbeds.^7^, ^8^, ^9^ Currently, commercial indoor tanning is legally available in the UK for those aged 18 years and older.

Successful implementation of the ban in Australia was partly attributable to accompanying public health advocacy.^10^ Similarly, a public information campaign about the health risks of indoor tanning and possible alternatives would maximize the likelihood of the success of bans elsewhere. Thus, a potentially effective policy‐based approach involves a ‘complex intervention’^11^ that encourages positive behaviour change in erstwhile sunbed users. A key consideration for governments is whether such a policy‐based intervention would represent value for money from the perspective of the healthcare system. The aim of this study was to determine the cost‐effectiveness of a policy‐based intervention to reduce the incidence of cutaneous melanoma and KC by banning exposure to commercial sunbeds in England.

## Materials and methods

We used a decision model‐ based cost‐effectiveness analysis to address the defined decision problem (Table 1). The study is reported in line with CHEERS^12^ criteria (Appendix S1; see Supporting Information). The study did not require ethical approval because data were assimilated from existing sources.

**Table 1:** Key design criteria

Decision problem	What are the incremental costs and consequences and key drivers of the relative cost‐effectiveness of a policy‐based complex intervention to reduce instances of skin cancer?
Intervention	Public health campaign and widespread ban on the provision of sunbeds in commercial settings in England
	A multimedia (including social media, radio and television) public health campaign would highlight the risks of indoor tanning, targeting 18‐year‐olds to inform people about the ban, and promote alternatives to the use of sunbeds
Comparator	The comparator is the current situation; sunbeds can be provided for use by businesses in England
Population	Potential users of commercial sunbeds who were aged 18 years living in England
Model type	Cohort‐based decision tree linked to a state‐transition Markov model (‘Markov model’)
Software	Excel 2016
Time horizon	Lifetime (to a maximum of 100 years): to reflect the long‐term consequences of using sunbeds and impact on morbidity and mortality from cutaneous melanoma and/or keratinocyte cancer
Cycle length (total number of cycles)	1 year: (83 total cycles), half‐cycle corrections used
Discounting	3.5% for both costs and consequences to be consistent with published NICE recommendations^a^
Study perspective	National Health Service (NHS) in England
Costs	National currency (£) at 2019 prices^b^
Consequences	Quality‐adjusted life‐years (QALYs)
Uncertainty	Deterministic: one‐way sensitivity analysis; two‐way sensitivity analysis; scenario analyses
	Probabilistic sensitivity analysis
Cost‐effectiveness threshold	NICE recommended threshold^a^ of £20 000 to £30 000 per QALY gained

NICE, National Institute for Health and Care Excellence. ^a^Methods guide for technology appraisal. ^b^Unit costs were inflated to 2019 prices where appropriate, using linear regression based on previous NHS cost increases (https://nhsprocurement.org.uk/health‐sector‐cost‐index‐update).

### Intervention and comparator

The target population comprised young people eligible to use commercially available sunbeds; we focused on the cohort of all 18‐year‐olds residing in England in 2019.^13^ The implementation of a nationwide ban on commercial indoor tanning combined with a public information campaign (i.e. the policy‐based intervention) was compared with the status quo of widespread availability of commercial indoor tanning in England (Table 1).^14^

### Model

The decision model structure was conceptualized by following published guidelines^15^ and represented the costs and consequences for the defined study cohort. A decision tree was linked to a state‐transition Markov model (hereafter Markov model), informed by previous work^16^ and supported by a rapid review of published economic analyses^16^, ^17^, ^18^, ^19^, ^20^, ^21^ and advice from three experts (an epidemiologist, an oncologist, a dermatologist). The decision tree (Appendix S2; see Supporting Information) captured the problem of maintaining the current availability of sunbeds vs. removing them from commercial availability.

**Figure 1:**
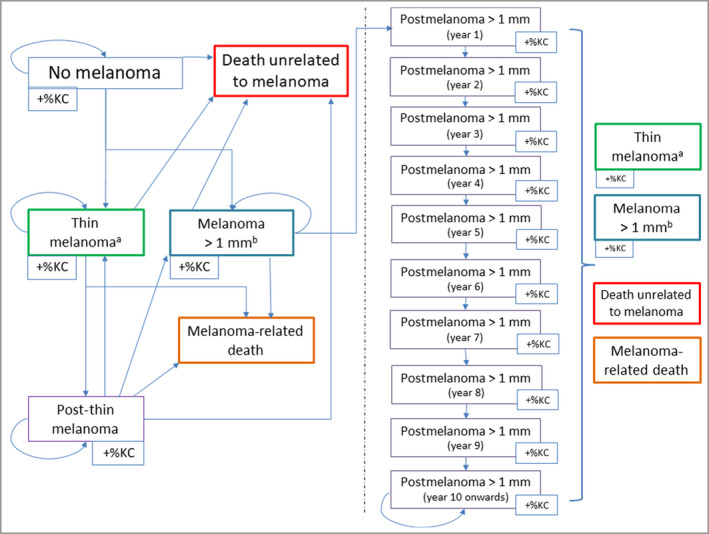
Diagrammatic representation of the Markov model. The six health states are as follows: no melanoma, death unrelated to melanoma (death from other causes), death related to melanoma, thin melanoma (thickness of ≤1 mm), thick melanoma (thickness of >1 mm), post‐thin melanoma (living with an increased mortality risk). Post‐thick melanoma is a tunnel state that follows an individual in the cohort from a diagnosis of thick melanoma (at year 1) for 10 years. +%KC indicates the probability of getting a keratinocyte cancer in any of the illustrated health states. Arrows indicate the possible routes through the model until death for an individual in the cohort. ^a^ *In situ* and stage 1. ^b^Stage 2, 3 and 4. [Colour figure can be viewed at wileyonlinelibrary.com]

The Markov‐model component (Figure 1) was used to represent the natural history of the chance of developing melanoma or a KC. It included six health states and 10 ‘tunnel’ states, the latter allowing inclusion of annual mortality risk for 10 years following diagnosis of higher‐risk melanoma >1‐mm thick. The proportion of individuals in the cohort who may develop KC was also represented. The decision model was a cohort‐based model. This entailed defining a starting age for the cohort that is relevant to the decision problem (here, 18 years of age). The cohort model then follows an 18‐year‐old through to death, consistent with the assumed time horizon for the model. Death can occur because of mortality from melanoma or another cause (Table 2).

**Table 2:** Model input parameters

Parameter	Base case value	Distribution	Mean (alpha)	SE (beta)	95% Confidence interval	Source
Probabilities and risks						
Nonmelanoma mortality^a^	^a^See Appendix S3	NA	NA	NA	NA	www.nomisweb.co.uk,^22^ Forman^23^
Prevalence of sunbed use^b^ (18‐year‐old male)	0.02	NA	See Appendix S3 and Appendix S5			Authors’ age/sex‐specific estimates of ‘ever‐use’ by cohort	
Prevalence of sunbed use^b^ (18‐year‐old female)	0.043	NA	See Appendix S3 and Appendix S5			Authors’ age/sex‐specific estimates of ‘ever‐use’ by cohort	
Time from exposure to sunbed to diagnosis (years)	9	Normal	9	0.9	7.24–10.76	Cust *et al*.^40^
Probability of melanoma in population (18‐year‐old female)^c^	0.00005	See Appendix S3	NA	NA	NA	Forman *et al*.^23^
Probability of keratinocyte cancer in population (18‐year‐old female)^d^	0.00001	See Appendix S3	NA	NA	NA	Venables *et al*.^26^
Relative risk: melanoma with sunbed use^e^	1.59	Lognormal	1.59	0.09	1.36–1.87	Boniol *et al*.^3^
Relative risk: keratinocyte cancer with sunbed use^f^	1.48	Lognormal	1.48	0.14	1.21–2.08	Wehner *et al*.^4^
Probability of melanoma >1 mm	0.354	Beta	(28.3)	(51.7)	0.253–0.461	Sacchetto *et al*.^41^
Mortality risk following melanoma >1 mm (year 1)^g^	0.0555	Normal	0.0555	0.00555	0.0446–0.0664	Authors’ estimate^24^
Mortality risk following melanoma >1 mm (≥ year 10)^h^	0.0056	NA	NA	NA	NA	Authors’ estimate^24^
Mortality risk following thin melanoma (increased mortality lifetime risk)^i^	0.0056	NA	NA	NA	NA	Authors’ estimate^24^
Utilities						
No melanoma (18‐year‐old)^j^	0.929	See Appendix S3	NA	NA	NA	Janssen *et al*.^28^
Thin melanoma^k^	0.93^l^	Normal	0.93	See Appendix S8		Wilson *et al*.^29^	
Keratinocyte cancer^k^	0.93	Normal	0.93	See Appendix S8		Authors’ assumption^m^	
Melanoma >1 mm^k^	0.837^n^	Normal	0.84	See Appendix S8		Wilson *et al*.^29^	
Costs						
Keratinocyte cancer treatment^o^	£1348	Normal	£1058^p^	£106^p^	£850–£1265^p^	Vallejo‐Torres *et al*.^42^
Thin melanoma treatment^o^	£1338	See Appendix S7	£1338	See Appendix S7		Wilson *et al*.^29^	
Melanoma >1 mm treatment^o^	£3182	See Appendix S7	£3182	See Appendix S7		Wilson *et al*.^29^	
Nonmelanoma death	£0	NA	NA	NA	NA	Authors’ assumption
Melanoma death^o^	£4686	Normal	£4265^p^	£427^p^	£3429–£5101^p^	Wilson *et al*.^29^
Campaign cost^o^	£1 000 000	Beta	2	5	NA	Stoptober annual costs 2019 and 2016^q^ (min £0, max £3.34 million)

NA, not applicable, not included in probabilistic sensitivity analysis. ^a^Age/sex‐specific based on all‐cause mortality (UK data). Adjusted to reflect death not related to melanoma. ^b^The following four components varied in probabilistic sensitivity analysis: (i) a female to male ratio of indoor tanning, (ii) an initial (for 18‐year‐olds) prevalence based on previous use, (iii) an initial yearly incidence rate and (iv) a year‐on‐year decline in the incidence rate. ^c^Age/sex‐specific cancer registry data adjusted to reflect melanoma not attributable to indoor tanning. ^d^Age/sex‐specific study data adjusted to reflect melanoma not attributable to indoor tanning. ^e^Increased risk applied to those who had ever used an indoor tanning device before the age of 35 years. ^f^Increased risk applied to those who had ever used an indoor tanning device. ^g^Initial starting point from which a yearly decrement of 0.0055 was subtracted for a period of 10 years to approximate available survival data. ^h^Final increased mortality risk resulting from yearly decrements applied to first‐year estimate (see previous footnote). This increased risk persisted for lifetime. ^i^Set equal to increased risk 10 years postmelanoma >1 mm for lifetime and added to background risk of nonmelanoma death. Assumed conservative estimate based on survival data suggesting a 5‐year risk of 0.03 and a 10‐year risk of 0.02. ^j^EuroQol‐5D population norms UK (England). ^k^Utility values used as a disutility multiplier to adjust age‐specific population norm values (e.g. 0.93 × population norm value for thin melanoma). ^l^Weighted average of utility values for *in situ* and stage 1 melanomas. ^m^Assumed to be the same as decrement for thin melanoma. ^n^Weighted average of utility values for stage 2, 3 and 4 melanomas. ^o^Inflated to 2019 prices (https://nhsprocurement.org.uk/health‐sector‐cost‐index‐update for treatment costs and https://www.in2013dollars.com/uk/inflation for campaign costs). ^p^Uninflated costs. ^q^https://assets.publishing.service.gov.uk/government/uploads/system/uploads/attachment_data/file/743814/Stoptober_2016_campaign_evaluation.pdf and https://assets.publishing.service.gov.uk/government/uploads/system/uploads/attachment_data/file/992284/Stoptober_2019_Evaluation.pdf.

**Table 3:** Main results for the deterministic analysis

Estimate	Current situation	Intervention	Difference: intervention compared with current situation
Number of melanoma cases^a^	25 116	23 910	−1206 (4.8%)
Number of melanoma deaths^a^	4478	4271	−207 (4.6%)
Number of KC cases^a^	122 441	118 454	−3987 (3.3%)
Total costs^a,b^	£41 618 865	£40 916 007	−£657 858
Total QALYs^a,b^	13 619 953	13 620 450	497 QALYs
ICER	–	Not applicable (intervention dominant)	–
Net health benefit^a,c^	–	530 QALYs	–
Incremental net benefit^a,d^	–	£10 599 040	–

KC, keratinocyte cancer; ICER, incremental cost‐effectiveness ratio; QALYS, quality‐adjusted life‐years. ^a^Based on a cohort of 618 873 18‐year‐olds living in England. ^b^Discounted at a rate of 3.5%. ^c^Net health benefit = incremental QALYs − (incremental costs/£20 000). ^d^Incremental net benefit = (£20 000 × incremental QALYs) − incremental costs.

**Table 4:** One‐way sensitivity analysis results

Model input parameter^a^	Assumed parameter value		Incremental cost per QALY gained		Incremental net benefit^b^	
	Worst‐case estimate	Best‐case estimate	Worst‐case estimate	Best‐case estimate	Worst‐case estimate	Best‐case estimate
Campaign cost	£3 339 807	£0	£3225	Not applicable: intervention dominant	£8 338 357	£11 565 224
Sunbed use: current situation	Low^c^	High^d^	£2173	Not applicable: intervention dominant	£3 352 377	£20 739 271
Relative risk of melanoma	1.36	1.85	Not applicable: intervention dominant	Not applicable: intervention dominant	£6 832 690	£14 855 198
First year mortality risk: melanoma > 1 mm	0.0446	0.0664	Not applicable: intervention dominant	Not applicable: intervention dominant	£9 037 351	£12 081 521
Proportion of melanomas > 1 mm	0.25	0.46	Not applicable: intervention dominant	Not applicable: intervention dominant	£9 176 790	£12 118 340
Sunbed use: intervention	0.03	0.01	Not applicable: intervention dominant	Not applicable: intervention dominant	£9 217 607	£11 980 555
Relative risk of keratinocyte cancer	1.21	2.08	Not applicable: intervention dominant	Not applicable: intervention dominant	£9 500 199	£13 040 908
Disutility multiplier: keratinocyte cancer	0.96	0.90	Not applicable: intervention dominant	Not applicable: intervention dominant	£10 320 841	£10 877 239
Treatment cost: keratinocyte cancer	£1083.66	£1612.00	Not applicable: intervention dominant	Not applicable: intervention dominant	£10 414 710	£10 783 370
Disutility multiplier: melanoma > 1 mm	0.91	0.77	Not applicable: intervention dominant	Not applicable: intervention dominant	£10 498 111	£10 699 969
Disutility multiplier: thin melanoma	0.96	0.90	Not applicable: intervention dominant	Not applicable: intervention dominant	£10 530 353	£10 667 727
Treatment cost: melanoma > 1 mm	£2558.36	£3805.68	Not applicable: intervention dominant	Not applicable: intervention dominant	£10 543 620	£10 654 460
Treatment cost: thin melanoma	£1075.81	£1600.32	Not applicable: intervention dominant	Not applicable: intervention dominant	£10 555 515	£10 642 565
Cost of death: melanoma	£3767.19	£5603.89	Not applicable: intervention dominant	Not applicable: intervention dominant	£10 564 008	£10 634 071
Male : female ratio	1.3	2.9	Not applicable: intervention dominant	Not applicable: intervention dominant	£10 576 425	£10 611 933

QALY, quality‐adjusted life‐year. ^a^ Appendix S9 (see Supporting Information) describes how the assumed values for best and worst case estimates were generated. ^b^Incremental net benefit = (£20 000 × incremental QALYs) − incremental costs. ^c^e.g. 0.0098 for an 18‐year‐old woman. ^d^e.g. 0.0869 for an 18‐year‐old woman.

#### Mortality

Published sex‐specific mortality data for England^13^ were used to calculate the annual probabilities of dying from causes unrelated to melanoma, by subtracting the risk of dying from melanoma from all‐cause mortality estimates. For those with melanoma, an increased mortality risk was applied to the baseline population risk^22^, ^23^ using available epidemiological data.^24^, ^25^ For those with melanoma >1 mm, the increased yearly risk of melanoma death, starting at 0.06 in the year of diagnosis, was diminished annually to 0.006 in the 10th year and then persisted over the individual’s lifetime. For those with thin melanoma (≤ 1 mm), an increased annual lifetime risk was applied based on published survival data.^24^

#### Prevalence of sunbed use

Age‐ and sex‐specific prevalence of sunbed use (proportion of the population who have ever used an indoor tanning device)^14^ was calculated for the cohort (Appendices S3–S5; see Supporting Information). To reflect possible continued sunbed use in noncommercial settings postintervention, we assumed a use prevalence of 2% after the ban.

#### Skin cancer incidence and increased risk attributable to sunbed use

The proportion of melanomas attributable to sunbeds estimated in a meta‐analysis^3^ was subtracted from registered melanoma cases to determine annual sex‐specific probabilities of being diagnosed with melanoma not attributable to sunbed use. Data from a UK cohort study^26^ were used to determine the annual age‐ and sex‐specific probabilities of developing a KC (either BCC or SCC). The proportion of KCs attributable to sunbed use^4^ was subtracted from these probabilities to determine an annual probability of KC not attributable to sunbed use. The pooled risk estimate of melanoma from the meta‐analysis [1.59, 95% confidence interval (CI) 1.36–1.85]^3^ was assigned to the proportion in each cohort who had used sunbeds before the age of 35. Sunbed users were deemed to have increased risk of KCs (1.48, 95% CI 1.21–2.08) in line with meta‐analysis findings.^4^

#### Costs

Healthcare resource use and associated unit costs were calculated in keeping with the study perspective (Table 1) whereby the cost of the public health campaign component of the intervention was covered by NHS England. Campaign costs were derived using information on amounts spent on a previous smoking cessation campaign (Appendix S6; see Supporting Information). Published cost estimates capturing the diagnosis, treatment and monitoring of patients with melanoma were sourced from a rapid review of the literature (Appendix S7; see Supporting Information) that identified previous UK‐based economic evaluations.^4^

#### Consequences

Health consequences were measured using quality‐adjusted life‐years (QALYs), the product of additional years of life multiplied by the health‐related quality of life (HRQoL) in those additional years. An underlying (baseline) HRQoL, measured by the EuroQol five‐dimensional three‐level (EQ‐5D‐3L) descriptive system,^27^ was applied to the cohort, taking age into account (age‐specific HRQoL). Population norms^28^ for EQ‐5D‐3L scores in England were used to reflect HRQoL for the proportion of the cohort in lesion‐free and postmelanoma states. Age‐specific norms for HRQoL were then adjusted for each relevant state in the Markov model. Previously estimated HRQoL values^29^ were used to produce weighted average multiplier decrements to adjust HRQoL for melanoma (≤ 1 mm, > 1 mm) and KC states (Appendix S8; see Supporting Information). For the proportion of the cohort affected by both melanoma and KC in one model cycle, only one adjustment (for the larger effect) was applied. A HRQoL score of zero was applied to the proportion of the cohort in death states.

### Main analysis

Numbers of cases of melanoma and KC averted and melanoma‐related deaths avoided in the intervention arm compared with the current situation (control arm) were calculated. An incremental analysis compared the difference in total expected costs and expected QALYs generated in the intervention and control arms. If the incremental expected costs and QALYs are positive, an incremental cost per QALY gained [incremental cost‐effectiveness ratio (ICER)] should be calculated using the formula:

$$\text{ICER}=\frac{\left(C_{2}\;-\;C_{1}\right)}{\left({\text{QALY}}_{2}\;-\;{\text{QALY}}_{1}\right)}$$

where ${\text{QALY}}_{2}$ and ${\text{QALY}}_{1}$ are the total QALYs generated by intervention and current situation, respectively; $C_{2}$ and $C_{1}$ are the total healthcare costs generated by intervention and current situation, respectively. This ICER was compared with the current threshold of acceptability used in the context of the NHS in England (£20 000 to £30 000). It is not necessary to calculate an ICER if the intervention produced additional benefits for reduced costs, because the intervention is said to dominate its comparator and is, consequently, deemed a good use of healthcare resources. Net monetary benefit (NMB) (net benefit measured in cost compared with the lower £20 000 bound of the threshold) and net health benefit (NHB) (net gain in QALYs compared with the lower £20 000 bound of the threshold)^30^ of the intervention were calculated using the lower £20 000 bound of the threshold range.

#### Sensitivity analysis

The following three deterministic sensitivity analyses were used to understand the key drivers of cost‐effectiveness: one‐way sensitivity analysis [model input parameters (Table 2) varied one at a time], two‐way sensitivity analysis (input parameters varied two at a time) and scenario analyses (analysis run using different assumptions). A probabilistic sensitivity analysis (PSA) was used to identify the joint effect of varying defined parameters simultaneously.^31^ A PSA involves running the decision model a number of times (iterations, here set at 5000) and calculating a series of expected costs and QALYS for each intervention based on model input values from specified ranges and distributions (Appendix S9; see Supporting Information).

In the one‐way sensitivity analysis, single predefined parameters of interest (Appendix S10; see Supporting Information) were varied one at a time using extreme bounds of plausible values. In the two‐way sensitivity analysis (Appendix S11; see Supporting Information), treatment costs (up to 200% of base case value, using 20% increments) and treatment effects (up to 50% of base‐case value, using 5% increments) were varied in combination. This sensitivity analysis was based on the knowledge that newer, more expensive treatments for advanced melanoma and adjuvant therapies^32^ have been approved for use in the UK (current estimates not publicly available).

Four scenario analyses were conducted to account for potential negative effects of removing sunbeds, lower melanoma risk from sunbed use, different public health campaign costs and increased average costs of treating melanoma >1 mm. A potential negative health effect (anxiety) of removing the availability of sunbeds was explored by using a utility decrement multiplier (range 0–10%). The cutoff point at which the NMB gained fell below £0 was calculated to show the maximum amount of disutility (i.e. perceived harm from denial of use) needed to question the potential cost‐effectiveness of the intervention. The assumed summary relative risk of melanoma was changed in the main analysis from 1.59 to 1.2^3^ and applied to all sunbed users. The effect of assuming different campaign costs was tested by systematically varying this parameter to determine the price at which the intervention would become cost‐neutral (zero incremental costs between the intervention and current practice).

## Results

There were 618 873 adults (51% of whom were male) aged 18 years residing in England in 2019, with one estimated commercial sunbed in operation for every 2954 residents.^7^ When compared with the current availability of sunbeds over the lifetime of this cohort, the introduction of the policy‐based intervention would generate reductions of 4.8% in melanoma cases (*n* = 1206), 4.6% in melanoma deaths (*n* = 207) and 3.3% in numbers of KCs (*n* = 3987). These translate to an additional 497 QALYs with a cost‐saving to NHS England of £697 858, meaning that the intervention dominates the current situation comparator (Table 3) as it increases health and saves money. Based on the lower £20 000 bound of the threshold, the intervention would result in an incremental net benefit (INB) of £10.6 million and an NHB of 530 QALYs.^30^

### Sensitivity analysis

The PSA results indicated a low degree of parameter uncertainty (Appendix S9). At a cost‐effectiveness threshold of £20 000 per QALY, there is a 99% likelihood of the intervention being cost‐effective.

The one‐way sensitivity analysis demonstrated that extreme plausible worst‐case scenario values for each parameter of uncertainty (i.e. favouring the current situation) resulted in the intervention always being cost‐effective (Table 4). In the worst‐case scenario where public health campaign costs were set to £3.4 million, an additional QALY would be realized for a cost of £3225, substantially less than the lower bound of the £20 000 to £30 000 threshold range of cost‐effectiveness. If the lowest prevalence of sunbed use is assumed (e.g. 0.0098 and 0.0046 for an 18‐year‐old woman and man, respectively), a cost of £2173 per QALY gained would be realized. For all other one‐way sensitivity analysis inputs, the policy‐based intervention remained dominant in each worst‐case scenario (Table 4).

The two‐way sensitivity analysis (Appendix S11; see Supporting Information) showed that the main analysis was robust to combining treatment costs and associated melanoma treatment effectiveness. The intervention would remain the dominant option with any combination of the prespecified increases in treatment costs and effects. If base‐case treatment costs were increased by 20%, with a resultant 50% reduced mortality from thicker melanomas, an INB of £6.5 million would be realized. If a 200% increase in base‐case treatment costs for thin melanomas with a modest 5% reduction in their mortality is assumed, the INB would be just over £10.75 million (Appendix S11).

The effect of applying a disutility multiplier to capture negative (anxiety) consequences of sunbed removal (Appendix S12; see Supporting Information) had to be a 7.5% decrement (0.925 *HRQoL) to call into question the cost‐effectiveness of the intervention. Where a lower summary relative risk value of 1.2^3^ was applied to all sunbed users, the intervention remained the dominant option generating an INB of £4.97 million. The intervention would be cost‐neutral with the current situation if the public health campaign costs were set at £1.69 million. Campaign costs would need to be set at more than £11.97 million to generate an additional cost per QALY gained greater than £20 000 per QALY.

## Discussion

To reduce harm from indoor tanning, a policy‐based intervention involving a nationwide ban on commercial indoor tanning and a public health campaign is highly likely to be a good use of healthcare resources from the perspective of NHS England. Previous economic evaluations have demonstrated how other policy‐based interventions capable of effecting change at the population level, such as the taxation of sugary foods and beverages, or raising the legal age of smoking, are typically an efficient use of resources.^33^, ^34^ If NHS England invested in a public health campaign to support the ban on sunbeds, we estimate that melanoma and KC burden would be reduced, NHS resources would be saved and deaths averted. Key drivers of cost‐effectiveness are the estimated prevalence of sunbed use and public health campaign cost. Sensitivity analyses demonstrated the robustness of these findings.

Our results reflect those from previous explorations of the effect of such legislation on healthcare systems and productivity in America, Europe and Australia,^16^, ^20^ and therefore add to the growing body of evidence supporting a ban on commercial sunbeds. We used a structured and transparent approach to assimilating all available data to understand the economic impact of banning sunbeds alongside a public health campaign funded by NHS England. We quantified the potential effect of uncertainty in input values and key assumptions in sensitivity analyses. For each assumption, a conservative approach was employed favouring the current situation rather than the intervention.

The key limitation of this study was our reliance on publicly available data that may not reflect current clinical and tanning practices. While robust estimates for model input values could be identified in the published literature, this was not true for costs and clinical effectiveness of current diagnosis, treatment and monitoring options for patients with melanoma.^35^ Advanced melanoma treatments have increased dramatically in the last 10 years and adjuvant treatment of stage 3 melanoma has been approved, which suggests that our base‐case values are underestimated. Clinical trials are under way in patients with stage 2 disease, a much larger patient population than stages 3 or 4. Capturing the true cost of drug acquisition and administration, toxicity effects and patient follow‐up is not unique to skin cancer, but rather reflects the fast pace of technological development in cancer care generally and the challenge to collect these data.^32^, ^36^

The sex‐specific use of sunbeds was estimated using a conservative value (female : male ratio = 1.76: 1) favouring the status quo. This value, based on plausible published estimates, likely understates the proportion of female users in England, and a less conservative value would make the intervention appear even more cost‐effective.

Results from the meta‐analysis^3^ used here and in previous economic evaluations have recently been questioned,^37^ in particular the assumption about ‘ever‐use’ and the metric for first use at younger ages owing to potential heterogeneity in the definition of ‘younger age’. We took a conservative approach in this respect by applying an increased risk to only the proportion of the cohort who first used an indoor tanning device before the age of 35 years, noting that these risk estimates took account of confounding factors (e.g. outdoor tanning). The robustness of the results was further demonstrated in the sensitivity analysis using the lower bound of the plausible range of relative risk estimates from the meta‐analysis^3^ and in the scenario where the lower summary relative risk from that study was applied; the intervention remained the dominant option in both.

We assumed no effect on HRQoL for the postmelanoma states in this analysis. There is some evidence, for example, that fear of cancer recurrence is a measurable phenomenon in cancer survivors generally,^38^ and in people with a personal history of melanoma specifically.^39^ If these utility decrements had been included in the model, the estimated QALYs gained from removing sunbeds would appear even larger than those currently estimated.

In the hypothetical scenario presented, all intervention costs were assigned to the public health campaign component of the intervention. An alternative use of resources would be to fund a ‘sunbed buy‐back’ scheme^7^ to encourage commercial sunbed providers to repurpose their businesses. In a situation where an additional £10.97 million in intervention costs were available for this purpose, each provider in England could be paid £3709 per premise;^7^ alongside the inclusion of a £1 million public health campaign (total intervention costs £11.97 million), this would remain a cost‐effective use of NHS resources.

In conclusion, this study provides evidence that introducing a ban on indoor tanning with a supporting public health campaign in England is cost‐saving from an NHS perspective, resulting in health gain for a population of 18‐year‐olds. In view of these findings and the potential to reduce harm, the implementation of a ban on the provision of indoor tanning should be given serious consideration.

## Author contributions

**Martin Eden:** Conceptualization (equal); data curation (equal); formal analysis (equal); investigation (equal); methodology (equal); software (equal); validation (equal); visualization (equal); writing – original draft (equal); writing – review and editing (equal). **Rob Hainsworth:** Conceptualization (equal); data curation (equal); formal analysis (equal); investigation (equal); methodology (equal); software (equal); validation (equal); visualization (equal); writing – original draft (equal); writing – review and editing (equal). **Louisa Gordon:** Conceptualization (equal); data curation (equal); funding acquisition (equal); methodology (equal); validation (equal); visualization (equal); writing – review and editing (equal). **Tracy Epton:** Conceptualization (equal); funding acquisition (equal); supervision (equal); writing – review and editing (equal). **Paul Lorigan:** Conceptualization (equal); funding acquisition (equal); methodology (equal); supervision (equal); validation (equal); writing – review and editing (equal). **Lesley Elizabeth Rhodes:** Conceptualization (equal); funding acquisition (equal); writing – review and editing (equal). **Richard Marais:** Conceptualization (equal); funding acquisition (equal); supervision (equal); writing – review and editing (equal). **Adele Green:** Conceptualization (equal); funding acquisition (equal); methodology (equal); supervision (equal); validation (equal); visualization (equal); writing – original draft (equal); writing – review and editing (equal). **Katherine Payne:** Conceptualization (equal); formal analysis (equal); funding acquisition (equal); investigation (equal); methodology (equal); supervision (equal); validation (equal); visualization (equal); writing – original draft (equal); writing – review and editing (equal).

## Supporting information


**Appendix S1** CHEERS checklist
**Appendix S2** Decision tree
**Appendix S3** Age‐ and sex‐specific input parameters
**Appendix S4** Identifying the evidence to estimate the prevalence of indoor tanning
**Appendix S5** Calculating the prevalence of age‐related and sex‐specific indoor tanning use
**Appendix S6** Identification of costs of the intervention
**Appendix S7** Identifying the costs of treating skin cancer
**Appendix S8** Utility multiplier decrements
**Appendix S9** Probabilistic sensitivity analysis
**Appendix S10** One‐way sensitivity analysis
**Appendix S11** Two‐way sensitivity analysis
**Appendix S12** Scenario analysis: applying disutility for denial of sunbed use.

**Video S1** Author video.
