# Supplementary material for: Cost‐effectiveness of a policy‐based intervention to reduce melanoma and other skin cancers associated with indoor tanning
Source: Br J Dermatol. 2022 May 18;187(1):105–14. doi: 10.1111/bjd.21046 (PMC9541204; doi:10.1111/bjd.21046)
Supplement: Supplementary file 1 — Appendix S1 CHEERS checklist Appendix S2 Decision tree Appendix S3 Age‐ and sex‐specific input parameters Appendix S4 Identifying the evidence to estimate the prevalence of indoor tanning Appendix S5 Calculating the prevalence of age‐related and sex‐specific indoor tanning use Appendix S6 Identification of costs of the intervention Appendix S7 Identifying the costs of treating skin cancer Appendix S8 Utility multiplier decrements Appendix S9 Probabilistic sensitivity analysis Appendix S10 One‐way sensitivity analysis Appendix S11 Two‐way sensitivity analysis Appendix S12 Scenario analysis: applying disutility for denial of sunbed use. [file BJD-187-105-s001.docx]

# Supplementary appendices for:

Eden M, Hainsworth R, Gordon L, Epton T, Lorigan P, Rhodes LE, Marais R, Green AC, Payne K. Cost-effectiveness of a policy-based intervention to reduce instances of skin cancer attributable to indoor tanning

**Appendix 1:** CHEERS checklist

**Appendix 2:** Decision tree

**Appendix 3**: Age and gender specific input parameters

**Appendix 4:** Identifying the evidence to estimate the prevalence of indoor tanning

**Appendix 5:** Calculating the prevalence of age-related and gender-specific indoor tanning use

**Appendix 6:** Identification of costs of the intervention

**Appendix 7:** Identifying the costs of treating skin cancer

**Appendix 8:** Utility multiplier decrements

**Appendix 9:** Probabilistic sensitivity analysis

**Appendix 10:** One-way sensitivity analysis

**Appendix 11:** Two-way sensitivity analysis

**Appendix 12:** Scenario analysis: applying disutility for denial of sunbed use

**Appendix 1:** CHEERS checklist

| Section/item | Item No | Recommendation | Reported on page No/ line No |
| --- | --- | --- | --- |
| **Title and abstract** | | | |
| Title | 1 | Identify the study as an economic evaluation or use more specific terms such as “cost-effectiveness analysis”, and describe the interventions compared. | Title page |
| Abstract | 2 | Provide a structured summary of objectives, perspective, setting, methods (including study design and inputs), results (including base case and uncertainty analyses), and conclusions. | Abstract |
| **Introduction** | | | |
| Background and objectives | 3 | Provide an explicit statement of the broader context for the study. | Pages 4 to 5 |
|  |  | Present the study question and its relevance for health policy or practice decisions. | Pages 4 to 5 |
| **Methods** | | | |
| Target population and subgroups | 4 | Describe characteristics of the base case population and subgroups analysed, including why they were chosen. | Table 1 and page 6 |
| Setting and location | 5 | State relevant aspects of the system(s) in which the decision(s) need(s) to be made. | Table 1 and pages3, 6 |
| Study perspective | 6 | Describe the perspective of the study and relate this to the costs being evaluated. | Table 1 and page 7 |
| Comparators | 7 | Describe the interventions or strategies being compared and state why they were chosen. | Table 1 and pages 4, 5 |
| Time horizon | 8 | State the time horizon(s) over which costs and consequences are being evaluated and say why appropriate. | Table 1 |
| Discount rate | 9 | Report the choice of discount rate(s) used for costs and outcomes and say why appropriate. | Table 1 |
| Choice of health outcomes | 10 | Describe what outcomes were used as the measure(s) of benefit in the evaluation and their relevance for the type of analysis performed. | Table 1, Table 2, pages 7,8 and Appendix 9 |
| Measurement of effectiveness | 11a | *Single study-based estimates:*Describe fully the design features of the single effectiveness study and why the single study was a sufficient source of clinical effectiveness data. | Not applicable |
|  | 11b | *Synthesis-based estimates:*Describe fully the methods used for identification of included studies and synthesis of clinical effectiveness data. | Table 2 and pages 5,6,7 |
| Measurement and valuation of preference based outcomes | 12 | If applicable, describe the population and methods used to elicit preferences for outcomes. | Not applicable: from published sources |
| Estimating resources and costs | 13a | *Single study-based economic evaluation:*Describe approaches used to estimate resource use associated with the alternative interventions. Describe primary or secondary research methods for valuing each resource item in terms of its unit cost. Describe any adjustments made to approximate to opportunity costs. | Not applicable |
|  | 13b | *Model-based economic evaluation:*Describe approaches and data sources used to estimate resource use associated with model health states. Describe primary or secondary research methods for valuing each resource item in terms of its unit cost. Describe any adjustments made to approximate to opportunity costs. | Table 2, page 7 and Appendices 6&7 |
| Currency, price date, and conversion | 14 | Report the dates of the estimated resource quantities and unit costs. Describe methods for adjusting estimated unit costs to the year of reported costs if necessary. Describe methods for converting costs into a common currency base and the exchange rate. | Tables 1&2 and Appendices 6&7 |
| Choice of model | 15 | Describe and give reasons for the specific type of decision-analytical model used. Providing a figure to show model structure is strongly recommended. | Figure 1, page 6, and Appendix 2 |
| Assumptions | 16 | Describe all structural or other assumptions underpinning the decision-analytical model. | Figure 1, page 6, and Appendix 2 |
| Analytical methods | 17 | Describe all analytical methods supporting the evaluation. This could include methods for dealing with skewed, missing, or censored data; extrapolation methods; methods for pooling data; approaches to validate or make adjustments (such as half cycle corrections) to a model; and methods for handling population heterogeneity and uncertainty. | Tables 1&2, pages 5,6,7, and Appendices 3 to11 |
| **Results** | | | |
| Study parameters | 18 | Report the values, ranges, references, and, if used, probability distributions for all parameters. Report reasons or sources for distributions used to represent uncertainty where appropriate. Providing a table to show the input values is strongly recommended. | Tables 1,2&4, pages 5,6,7, and Appendices 3 to 11 |
| Incremental costs and outcomes | 19 | For each intervention, report mean values for the main categories of estimated costs and outcomes of interest, as well as mean differences between the comparator groups. If applicable, report incremental cost-effectiveness ratios. | Table 3, page 9 |
| Characterising uncertainty | 20a | *Single study-based economic evaluation:*Describe the effects of sampling uncertainty for the estimated incremental cost and incremental effectiveness parameters, together with the impact of methodological assumptions (such as discount rate, study perspective). | Not applicable |
|  | 20b | *Model-based economic evaluation:*Describe the effects on the results of uncertainty for all input parameters, and uncertainty related to the structure of the model and assumptions. | Table 4, pages 9,10, 11, Appendices 9 to 12 |
| Characterising heterogeneity | 21 | If applicable, report differences in costs, outcomes, or cost-effectiveness that can be explained by variations between subgroups of patients with different baseline characteristics or other observed variability in effects that are not reducible by more information. | Pages 10&11 and Appendix 12 |
| **Discussion** | | | |
| Study findings, limitations, generalisability, and current knowledge | 22 | Summarise key study findings and describe how they support the conclusions reached. Discuss limitations and the generalisability of the findings and how the findings fit with current knowledge. | Pages 10,11, 12, 13 |
| **Other** | | | |
| Source of funding | 23 | Describe how the study was funded and the role of the funder in the identification, design, conduct, and reporting of the analysis. Describe other non-monetary sources of support. | See source of funding section |
| Conflicts of interest | 24 | Describe any potential for conflict of interest of study contributors in accordance with journal policy. In the absence of a journal policy, we recommend authors comply with International Committee of Medical Journal Editors recommendations. | See Conflicts of interest section |

Source: Husereau D et al. Consolidated Health Economic Evaluation Reporting Standards (CHEERS) - explanation and elaboration: a report of the ISPOR Health Economic Evaluation Publication Guidelines Good Reporting Practices Task Force. *Value Health*. 2013;16(2):231-250

**Appendix 2:** Decision tree

This schematic highlights how a decision about whether or not to introduce the intervention will have an impact upon the proportion of the cohort that do or do not use sunbeds

Figure S2.1: decision tree

**
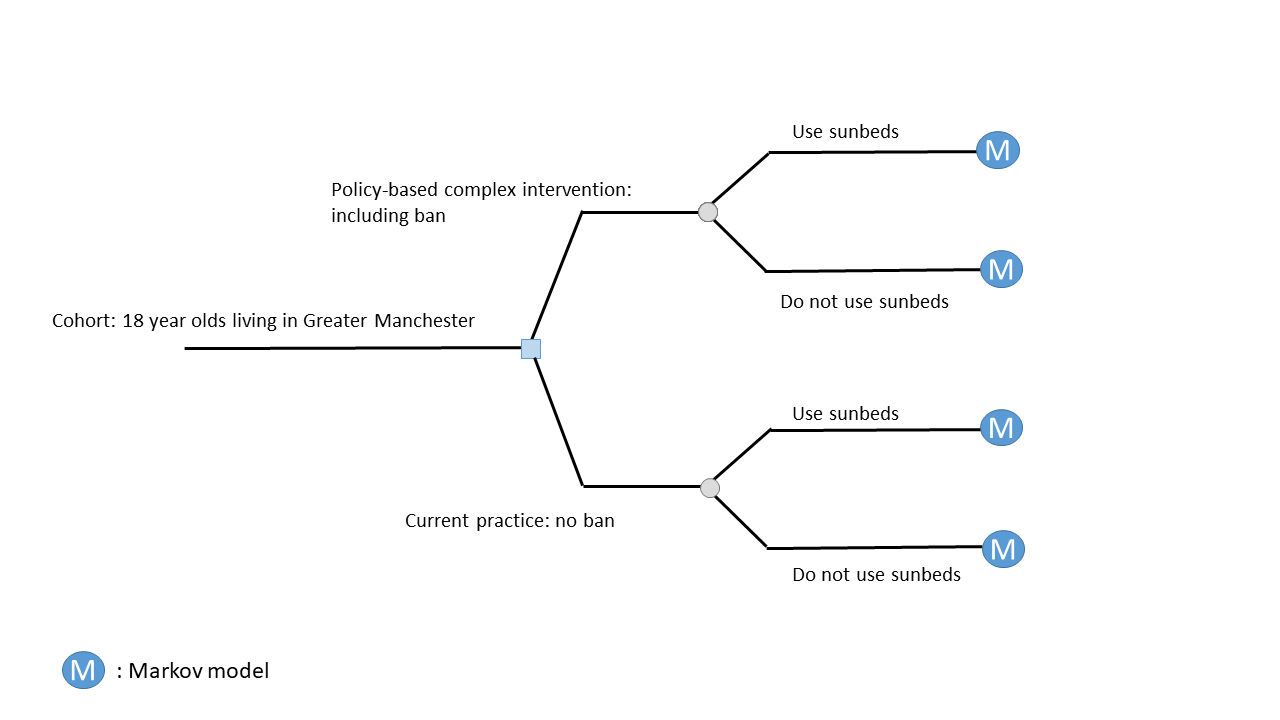
**

**Appendix 3:** Age and gender specific input parameters

Table S3.1 details individual values used in each cycle of the state transition Markov model based on age and gender.

Details for each parameter - including sources - are provided within the main article text and in Table 2.

Further detail on how sunbed use prevalence was calculated is provided in Appendix 4.

**Table S3.1: age and gender specific input parameters**

| **Age** | **Annual probability of death unrelated to melanoma (male)** | **Annual probability of death unrelated to melanoma (female)** | **Sunbed use prevalence**  **(male)** | **Sunbed use prevalence**  **(female)** | **Annual probability of thin melanoma without sunbed use**  **(male)** | **Annual probability of thin melanoma without sunbed use**  **(female)** | **Annual probability of melanoma >1mm without sunbed use**  **(male)** | **Annual probability of melanoma >1mm without sunbed use**  **(female)** | **Annual probability of KC without sunbed use**  **(male)** | **Annual probability of KC without sunbed use**  **(female)** | **Population norms for the EQ-5D** |
| --- | --- | --- | --- | --- | --- | --- | --- | --- | --- | --- | --- |
| 18 | 0.00037 | 0.00018 | 0.0204 | 0.0430 | 0.000019 | 0.000030 | 0.000011 | 0.000016 | 0.00001 | 0.00001 | 0.929 |
| 19 | 0.00042 | 0.00019 | 0.0265 | 0.0559 | 0.000024 | 0.000040 | 0.000013 | 0.000022 | 0.00001 | 0.00002 | 0.929 |
| 20 | 0.00046 | 0.00020 | 0.0321 | 0.0678 | 0.000028 | 0.000050 | 0.000015 | 0.000027 | 0.00002 | 0.00002 | 0.929 |
| 21 | 0.00047 | 0.00020 | 0.0373 | 0.0787 | 0.000033 | 0.000059 | 0.000018 | 0.000032 | 0.00002 | 0.00002 | 0.929 |
| 22 | 0.00049 | 0.00020 | 0.0420 | 0.0887 | 0.000037 | 0.000069 | 0.000020 | 0.000038 | 0.00002 | 0.00002 | 0.929 |
| 23 | 0.00050 | 0.00022 | 0.0464 | 0.0979 | 0.000045 | 0.000079 | 0.000025 | 0.000043 | 0.00002 | 0.00003 | 0.929 |
| 24 | 0.00051 | 0.00023 | 0.0504 | 0.1064 | 0.000053 | 0.000090 | 0.000029 | 0.000049 | 0.00003 | 0.00004 | 0.929 |
| 25 | 0.00053 | 0.00024 | 0.0541 | 0.1142 | 0.000060 | 0.000100 | 0.000033 | 0.000055 | 0.00004 | 0.00005 | 0.919 |
| 26 | 0.00056 | 0.00026 | 0.0575 | 0.1214 | 0.000068 | 0.000110 | 0.000037 | 0.000060 | 0.00005 | 0.00007 | 0.919 |
| 27 | 0.00059 | 0.00028 | 0.0607 | 0.1280 | 0.000075 | 0.000117 | 0.000041 | 0.000064 | 0.00007 | 0.00009 | 0.919 |
| 28 | 0.00062 | 0.00030 | 0.0636 | 0.1341 | 0.000080 | 0.000128 | 0.000044 | 0.000070 | 0.00009 | 0.00011 | 0.919 |
| 29 | 0.00067 | 0.00032 | 0.0662 | 0.1397 | 0.000085 | 0.000139 | 0.000047 | 0.000076 | 0.00010 | 0.00013 | 0.919 |
| 30 | 0.00071 | 0.00035 | 0.0686 | 0.1448 | 0.000090 | 0.000149 | 0.000050 | 0.000082 | 0.00012 | 0.00016 | 0.919 |
| 31 | 0.00075 | 0.00038 | 0.0709 | 0.1496 | 0.000096 | 0.000160 | 0.000052 | 0.000087 | 0.00014 | 0.00018 | 0.919 |
| 32 | 0.00080 | 0.00043 | 0.0730 | 0.1539 | 0.000101 | 0.000170 | 0.000055 | 0.000093 | 0.00015 | 0.00020 | 0.919 |
| 33 | 0.00085 | 0.00047 | 0.0749 | 0.1579 | 0.000111 | 0.000180 | 0.000061 | 0.000098 | 0.00018 | 0.00023 | 0.919 |
| 34 | 0.00091 | 0.00051 | 0.0766 | 0.1616 | 0.000121 | 0.000190 | 0.000066 | 0.000104 | 0.00021 | 0.00026 | 0.919 |
| 35 | 0.00100 | 0.00057 | 0.0782 | 0.1650 | 0.000131 | 0.000200 | 0.000072 | 0.000109 | 0.00025 | 0.00030 | 0.893 |
| 36 | 0.00106 | 0.00062 | 0.0797 | 0.1681 | 0.000141 | 0.000209 | 0.000077 | 0.000115 | 0.00029 | 0.00035 | 0.893 |
| 37 | 0.00114 | 0.00067 | 0.0810 | 0.1710 | 0.000151 | 0.000219 | 0.000083 | 0.000120 | 0.00034 | 0.00040 | 0.893 |
| 38 | 0.00123 | 0.00073 | 0.0823 | 0.1737 | 0.000162 | 0.000229 | 0.000089 | 0.000125 | 0.00039 | 0.00045 | 0.893 |
| 39 | 0.00133 | 0.00079 | 0.0835 | 0.1761 | 0.000173 | 0.000239 | 0.000094 | 0.000131 | 0.00043 | 0.00049 | 0.893 |
| 40 | 0.00142 | 0.00085 | 0.0845 | 0.1783 | 0.000183 | 0.000249 | 0.000100 | 0.000136 | 0.00048 | 0.00054 | 0.893 |
| 41 | 0.00156 | 0.00093 | 0.0855 | 0.1804 | 0.000194 | 0.000259 | 0.000106 | 0.000142 | 0.00052 | 0.00058 | 0.893 |
| 42 | 0.00169 | 0.00103 | 0.0864 | 0.1823 | 0.000204 | 0.000268 | 0.000112 | 0.000147 | 0.00056 | 0.00063 | 0.893 |
| 43 | 0.00185 | 0.00113 | 0.0872 | 0.1840 | 0.000213 | 0.000273 | 0.000116 | 0.000149 | 0.00063 | 0.00069 | 0.893 |
| 44 | 0.00200 | 0.00125 | 0.0880 | 0.1856 | 0.000221 | 0.000278 | 0.000121 | 0.000152 | 0.00070 | 0.00075 | 0.893 |
| 45 | 0.00218 | 0.00137 | 0.0887 | 0.1871 | 0.000229 | 0.000282 | 0.000125 | 0.000155 | 0.00078 | 0.00081 | 0.855 |
| 46 | 0.00235 | 0.00150 | 0.0893 | 0.1884 | 0.000238 | 0.000287 | 0.000130 | 0.000157 | 0.00087 | 0.00088 | 0.855 |
| 47 | 0.00257 | 0.00163 | 0.0899 | 0.1897 | 0.000246 | 0.000292 | 0.000135 | 0.000160 | 0.00096 | 0.00095 | 0.855 |
| 48 | 0.00277 | 0.00177 | 0.0904 | 0.1908 | 0.000259 | 0.000297 | 0.000142 | 0.000163 | 0.00105 | 0.00101 | 0.855 |
| 49 | 0.00301 | 0.00193 | 0.0909 | 0.1919 | 0.000273 | 0.000302 | 0.000150 | 0.000165 | 0.00114 | 0.00107 | 0.855 |
| 50 | 0.00325 | 0.00210 | 0.0914 | 0.1929 | 0.000287 | 0.000308 | 0.000157 | 0.000168 | 0.00123 | 0.00113 | 0.855 |
| 51 | 0.00351 | 0.00226 | 0.0918 | 0.1938 | 0.000300 | 0.000313 | 0.000164 | 0.000171 | 0.00132 | 0.00119 | 0.855 |
| 52 | 0.00376 | 0.00244 | 0.0922 | 0.1946 | 0.000314 | 0.000318 | 0.000172 | 0.000174 | 0.00140 | 0.00125 | 0.855 |
| 53 | 0.00404 | 0.00264 | 0.0926 | 0.1953 | 0.000333 | 0.000327 | 0.000183 | 0.000179 | 0.00154 | 0.00134 | 0.855 |
| 54 | 0.00438 | 0.00286 | 0.0929 | 0.1960 | 0.000353 | 0.000336 | 0.000193 | 0.000184 | 0.00172 | 0.00144 | 0.855 |
| 55 | 0.00476 | 0.00310 | 0.0932 | 0.1967 | 0.000372 | 0.000344 | 0.000204 | 0.000189 | 0.00193 | 0.00156 | 0.81 |
| 56 | 0.00520 | 0.00340 | 0.0935 | 0.1973 | 0.000391 | 0.000353 | 0.000214 | 0.000193 | 0.00217 | 0.00170 | 0.81 |
| 57 | 0.00568 | 0.00373 | 0.0937 | 0.1978 | 0.000411 | 0.000362 | 0.000225 | 0.000198 | 0.00244 | 0.00185 | 0.81 |
| 58 | 0.00623 | 0.00408 | 0.0940 | 0.1983 | 0.000446 | 0.000378 | 0.000244 | 0.000207 | 0.00270 | 0.00199 | 0.81 |
| 59 | 0.00680 | 0.00445 | 0.0942 | 0.1988 | 0.000481 | 0.000395 | 0.000263 | 0.000216 | 0.00296 | 0.00213 | 0.81 |
| 60 | 0.00745 | 0.00493 | 0.0944 | 0.1992 | 0.000516 | 0.000411 | 0.000282 | 0.000225 | 0.00322 | 0.00227 | 0.81 |
| 61 | 0.00819 | 0.00541 | 0.0946 | 0.1996 | 0.000551 | 0.000427 | 0.000301 | 0.000234 | 0.00348 | 0.00241 | 0.81 |
| 62 | 0.00898 | 0.00593 | 0.0948 | 0.1999 | 0.000586 | 0.000444 | 0.000321 | 0.000243 | 0.00374 | 0.00255 | 0.81 |
| 63 | 0.00987 | 0.00649 | 0.0949 | 0.2003 | 0.000617 | 0.000454 | 0.000338 | 0.000249 | 0.00410 | 0.00274 | 0.81 |
| 64 | 0.01084 | 0.00709 | 0.0950 | 0.2006 | 0.000648 | 0.000465 | 0.000355 | 0.000254 | 0.00451 | 0.00295 | 0.81 |
| 65 | 0.01186 | 0.00767 | 0.0952 | 0.2008 | 0.000679 | 0.000475 | 0.000372 | 0.000260 | 0.00498 | 0.00317 | 0.773 |
| 66 | 0.01296 | 0.00839 | 0.0953 | 0.2011 | 0.000710 | 0.000486 | 0.000389 | 0.000266 | 0.00549 | 0.00341 | 0.773 |
| 67 | 0.01420 | 0.00916 | 0.0954 | 0.2013 | 0.000741 | 0.000496 | 0.000406 | 0.000272 | 0.00606 | 0.00368 | 0.773 |
| 68 | 0.01539 | 0.01001 | 0.0955 | 0.2015 | 0.000775 | 0.000503 | 0.000424 | 0.000275 | 0.00658 | 0.00391 | 0.773 |
| 69 | 0.01678 | 0.01094 | 0.0956 | 0.2017 | 0.000809 | 0.000509 | 0.000443 | 0.000279 | 0.00710 | 0.00414 | 0.773 |
| 70 | 0.01829 | 0.01209 | 0.0957 | 0.2019 | 0.000843 | 0.000516 | 0.000461 | 0.000282 | 0.00762 | 0.00437 | 0.773 |
| 71 | 0.02022 | 0.01347 | 0.0958 | 0.2021 | 0.000876 | 0.000523 | 0.000480 | 0.000286 | 0.00813 | 0.00461 | 0.773 |
| 72 | 0.02229 | 0.01500 | 0.0959 | 0.2022 | 0.000910 | 0.000529 | 0.000498 | 0.000290 | 0.00865 | 0.00484 | 0.773 |
| 73 | 0.02482 | 0.01675 | 0.0959 | 0.2024 | 0.000936 | 0.000537 | 0.000512 | 0.000294 | 0.00929 | 0.00512 | 0.773 |
| 74 | 0.02772 | 0.01884 | 0.0960 | 0.2025 | 0.000961 | 0.000545 | 0.000526 | 0.000298 | 0.00996 | 0.00540 | 0.773 |
| 75 | 0.03099 | 0.02117 | 0.0960 | 0.2026 | 0.000987 | 0.000553 | 0.000540 | 0.000303 | 0.01066 | 0.00569 | 0.703 |
| 76 | 0.03450 | 0.02378 | 0.0961 | 0.2028 | 0.001012 | 0.000561 | 0.000554 | 0.000307 | 0.01137 | 0.00597 | 0.703 |
| 77 | 0.03844 | 0.02681 | 0.0961 | 0.2029 | 0.001038 | 0.000569 | 0.000568 | 0.000311 | 0.01211 | 0.00626 | 0.703 |
| 78 | 0.04294 | 0.03020 | 0.0962 | 0.2029 | 0.001063 | 0.000578 | 0.000582 | 0.000316 | 0.01275 | 0.00650 | 0.703 |
| 79 | 0.04785 | 0.03400 | 0.0962 | 0.2030 | 0.001089 | 0.000587 | 0.000596 | 0.000321 | 0.01338 | 0.00674 | 0.703 |
| 80 | 0.05328 | 0.03829 | 0.0963 | 0.2031 | 0.001114 | 0.000596 | 0.000610 | 0.000326 | 0.01402 | 0.00698 | 0.703 |
| 81 | 0.05945 | 0.04335 | 0.0963 | 0.2032 | 0.001139 | 0.000604 | 0.000624 | 0.000331 | 0.01466 | 0.00722 | 0.703 |
| 82 | 0.06676 | 0.04913 | 0.0963 | 0.2033 | 0.001165 | 0.000613 | 0.000638 | 0.000336 | 0.01529 | 0.00746 | 0.703 |
| 83 | 0.07482 | 0.05588 | 0.0964 | 0.2033 | 0.001192 | 0.000619 | 0.000652 | 0.000339 | 0.01599 | 0.00772 | 0.703 |
| 84 | 0.08410 | 0.06373 | 0.0964 | 0.2034 | 0.001219 | 0.000625 | 0.000667 | 0.000342 | 0.01662 | 0.00796 | 0.703 |
| 85 | 0.09455 | 0.07256 | 0.0964 | 0.2034 | 0.001246 | 0.000631 | 0.000682 | 0.000346 | 0.01719 | 0.00818 | 0.703 |
| 86 | 0.10621 | 0.08253 | 0.0964 | 0.2035 | 0.001272 | 0.000637 | 0.000697 | 0.000349 | 0.01769 | 0.00838 | 0.703 |
| 87 | 0.11915 | 0.09346 | 0.0965 | 0.2035 | 0.001299 | 0.000643 | 0.000711 | 0.000352 | 0.01813 | 0.00855 | 0.703 |
| 88 | 0.13212 | 0.10570 | 0.0965 | 0.2036 | 0.001299 | 0.000643 | 0.000711 | 0.000352 | 0.01844 | 0.00867 | 0.703 |
| 89 | 0.14656 | 0.11909 | 0.0965 | 0.2036 | 0.001299 | 0.000643 | 0.000711 | 0.000352 | 0.01875 | 0.00880 | 0.703 |
| 90 | 0.16212 | 0.13378 | 0.0965 | 0.2036 | 0.001299 | 0.000643 | 0.000711 | 0.000352 | 0.01906 | 0.00892 | 0.703 |
| 91 | 0.17860 | 0.14929 | 0.0965 | 0.2037 | 0.001299 | 0.000643 | 0.000711 | 0.000352 | 0.01937 | 0.00905 | 0.703 |
| 92 | 0.19635 | 0.16643 | 0.0965 | 0.2037 | 0.001299 | 0.000643 | 0.000711 | 0.000352 | 0.01968 | 0.00917 | 0.703 |
| 93 | 0.21681 | 0.18514 | 0.0965 | 0.2037 | 0.001299 | 0.000643 | 0.000711 | 0.000352 | 0.01999 | 0.00930 | 0.703 |
| 94 | 0.23896 | 0.20530 | 0.0966 | 0.2037 | 0.001299 | 0.000643 | 0.000711 | 0.000352 | 0.02023 | 0.00940 | 0.703 |
| 95 | 0.26044 | 0.22529 | 0.0966 | 0.2038 | 0.001299 | 0.000643 | 0.000711 | 0.000352 | 0.02042 | 0.00947 | 0.703 |
| 96 | 0.28221 | 0.24668 | 0.0966 | 0.2038 | 0.001299 | 0.000643 | 0.000711 | 0.000352 | 0.02054 | 0.00952 | 0.703 |
| 97 | 0.30870 | 0.26781 | 0.0966 | 0.2038 | 0.001299 | 0.000643 | 0.000711 | 0.000352 | 0.02060 | 0.00954 | 0.703 |
| 98 | 0.33355 | 0.29095 | 0.0966 | 0.2038 | 0.001299 | 0.000643 | 0.000711 | 0.000352 | 0.02060 | 0.00954 | 0.703 |
| 99 | 0.34488 | 0.30135 | 0.0966 | 0.2038 | 0.001299 | 0.000643 | 0.000711 | 0.000352 | 0.02060 | 0.00954 | 0.703 |
| 100 | 0.35899 | 0.31334 | 0.0966 | 0.2039 | 0.001299 | 0.000643 | 0.000711 | 0.000352 | 0.02060 | 0.00954 | 0.703 |

**Appendix 4:** Identifying the evidence to estimate the prevalence of indoor tanning

*Aim:* to identify the evidence to estimate the prevalence of indoor tanning in England

*Methods: identifying the evidence*

A rapid review was conducted in line with the definition provided by Kaltenhalter et al. (1). A two-stage approach was employed; first, a citation pearl-growing method (2) was used to identify studies and, secondly, relevant databases were interrogated using a set of pre-defined search terms (see below). The review was conducted in 2021.

*Pearl citation approach*

An economic evaluation with meta-analysis of sunbed use prevalence was used as the initial key text from which to proceed (2). Potentially useful references within this key text were identified by two researchers and full texts of these articles were retrieved. For those papers meeting the inclusion criteria (see below) the process of identifying relevant references within each was repeated.

*Database searches*

The following search string was used in the Pubmed and Web of Science databases: ‘indoor tanning’ or ‘sunbed’ or ‘tanning bed’ or ‘tanning booth’ or ‘tanning salon’ or ‘solarium’ or ‘solaria’ or ‘sunlamp’ or ‘artificial tanning’ or ‘UV tanning’ or ‘nonsolar ultraviolet radiation’ or ‘nonsolar UV radiation’. To maximise the relevance of the results, the searches were filtered for UK studies.

*Inclusion/exclusion criteria*

Studies were included if:

- quantified estimates of sunbed use were provided, or
- other evidence on sunbed usage (e.g. by age, sex or over time) was described, and
- the evidence was specifically related to the UK populations or could potentially be used in lieu of UK-specific data

*Study selection and data extraction*

Title and abstract screening was undertaken independently by two reviewers. Full text of papers were retrieved when it was unclear whether or not to include following abstract screening. For all included papers evidence on incidence, prevalence, trends, and demographic factors was extracted.

**Results**

Two papers were identified from the citation pearl-growing exercise and a further one paper from the database searchers. Following removal of duplicates, 155 papers were subject to title and abstracts screening. After screening, a total of 71 papers were included in the review and full text of each retrieved.

Including the key text used for pearl growing, four papers were selected to be used in calculating indoor tanning prevalence (see Table S4.1 and Appendix 4b below).

**Table S4.1: papers identified for use in sunbed use prevalence calculations**

| **Author (year)** | **Location** | **Data collection year(s)** | **Evidence** |
| --- | --- | --- | --- |
| Hirst et al. (2009) | Australia | NA | Change in incidence by age |
| Rodriguez Acevedo et al. (2017) | Global | 2007-2018 | Starting (18 year-old) prevalence, starting incidence, change in prevalence by calendar year |
| Suppa et al. (2019) | Europe | 2009-2014 | Starting prevalence, relative prevalence by gender, change in prevalence by calendar year |
| Gordon et al. (2020) | North America & Europe | NA | Key text, definitions of use |

1. Kaltenthaler E, Tappenden P, Paisley S, Squires H. Nice DSU Technical Support Document 13: Identifying and Reviewing Evidence To Inform the Conceptualisation and Population of Cost-Effectiveness Models. NICE DSU Technical Support Document. 2011.

2. Booth A. Unpacking your literature search toolbox: on search styles and tactics. *Health Info Libr J* 2008;**25**:313–7. doi:[10.1111/j.1471-1842.2008.00825.x](https://doi.org/10.1111/j.1471-1842.2008.00825.x)

**Appendix 5:** Calculating the prevalence of age-related and gender-specific indoor tanning use

*Aim:* to calculate an evidence-based, age-related and gender-specific prevalence of sunbed use for people aged 18 to 100 living in England

*Method*: Using the outputs of the rapid review of the literature reported in Appendix 4, an estimated prevalence of sunbed use at each age was calculated following the method reported in Hirst et al. (1) and using the following parameters:

- initial (for 18 year-olds) prevalence based on previous use
- year-on-year decline in sunbed prevalence
- initial yearly incidence rate
- rate of change of incidence wit years of age
- female-to-male ratio of sunbed use

Prevalence is defined as ‘ever use’ in keeping with previous economic evaluations (2). Incidence was therefore defined as first use in a year of age. Point estimates of sunbed usage from the European study (3) and Rodriguez-Acevedo et al.’s paper (4) were compared to estimate the rate of change in sunbed prevalence every two calendar years. Eighteen-year-old prevalence for 2020 was projected from the two studies using the two-yearly rate and fixed as a start-point (3). Incidence was assumed to fall by 8% with each year of age, following Hirst et al. (1).

Given the fixed starting prevalence and assumed rate of change in incidence with age, starting incidence (at eighteen years of age) was calibrated so that prevalence would peak at the point estimate of adult prevalence from Rodriguez-Acevedo et al.’s paper, adjusted to 2020 (4). Using the adult estimate as a maximum is likely to underestimate overall sunbed use, and therefore provide conservative estimates of the cost-effectiveness of a ban. A ratio of female-to-male use of 2.11 to 1 was used as reported in a published Irish sample of both adults and adolescents (3).

*Results:* An estimated ever-use prevalence of 0.0204 and 0.0430 was calculated for an 18 year-old male and female, respectively. By the age of 35, it was calculated that 0.0782 of the male cohort and 0.1650 of the female cohort would have used a sunbed. See Figure 1 and Table S2 in appendix 3 for sunbed use prevalence curve and age specific values.

**Figure S5.1: estimated sunbed use prevalence by age and gender**


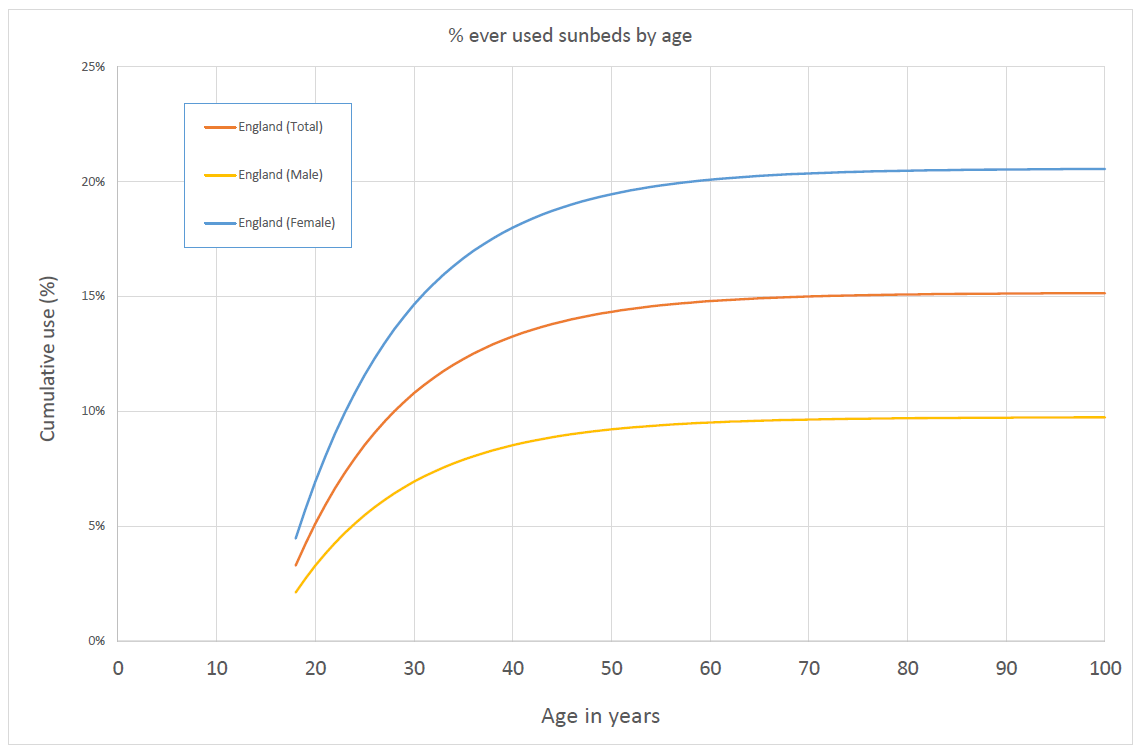


*References*

1. Hirst N, Gordon L, Gies P, Green AC. Estimation of avoidable skin cancers and cost-savings to government associated with regulation of the solarium industry in Australia. Health Policy. 2009;89(3):303-311. doi:10.1016/j.healthpol.2008.07.003

2. Gordon LG, Rodriguez-Acevedo AJ, Køster B, et al. Association of Indoor Tanning Regulations With Health and Economic Outcomes in North America and Europe. JAMA Dermatol. Published online February 19, 2020. doi:10.1001/jamadermatol.2020.0001

3. Suppa M, Gandini S, Njimi H, et al. Prevalence and determinants of sunbed use in thirty European countries: data from the Euromelanoma skin cancer prevention campaign. Journal of the European Academy of Dermatology and Venereology. 2019;33(S2):13-27. doi:10.1111/jdv.15311

4. Rodriguez‐Acevedo AJ, Green AC, Sinclair C, Deventer E van, Gordon LG. Indoor tanning prevalence after the International Agency for Research on Cancer statement on carcinogenicity of artificial tanning devices: systematic review and meta-analysis. British Journal of Dermatology. n/a(n/a). doi:10.1111/bjd.18412

**Appendix 6:** Identification of costs of the intervention

*Aim:* to determine a plausible estimate of costs attributable to the public health campaign aspect of the intervention

*Method:* A literature search was conducted to identify previously reported costs of public health campaigns undertaken in England. Where necessary, identified costs were inflated to reflect 2019 prices using an online inflation tool (https://www.in2013dollars.com/).

*Results*: An assumed one-off cost of £1million for the campaign element of the England-wide intervention was used. The figure is based on the annual amount spent on the ‘Stoptober’ smoking cessation campaign by Public Health England during 2019 (90). An upper limit to use in sensitivity analysis was based on the amount spent of the campaign during 2015 (the highest annual spend in previous years) adjusted to 2019 prices (https://www.in2013dollars.com/). A lower limit of £0 to be used in the sensitivity analyses reflects a situation in which the NHS would not bear the cost of a public health campaign.

**Appendix 7:** Identifying the costs of treating skin cancer

*Aim*: This study aimed to identify plausible costs associated with treating skin cancer.

*Methods*: A rapid review using an electronic search strategy. Results were limited to the years 2015 to 2020 to keep the costs relevant to current practice. The Medline database was interrogated in August 2020 using search terms detailed in table S6.1. Results from the PubMed database were limited to the last ten years.

*Inclusion criteria:*

- studies reporting annual UK treatment costs for melanoma or keratinocyte cancers
- *Exclusion criteria:*
- Review articles
- Non-English language

One reviewer (ME) did a) title, b) title & abstract and c) full text screening to identify potentially relevant papers

*Results:* Titles from an initial pool of 1203 studies were screened. Abstract screening was done on 65 papers and the full text of 16 papers was examined. Two additional papers identified during the full text screening were retrieved. Four papers were included in the final review (1, 2, 3, 4) along with three additional papers published before 2015 (5,6,7), which had been cited in four articles of interest (see Table S6.2)

The most recent data on treatment costs for melanoma was published by Wilson et al. (4|) in 2018 and the most recent data on treatment costs for non-melanoma skin cancer was published by Vallejo-Torres et al. (6) in 2014.

**Table S7.1: search terms to identify skin cancer treatment costs**

| **Search terms for skin cancer costs**  1. exp Health Care Costs/ |
| --- |
| 2. exp Health Expenditures/ |
| 3. cost.mp. |
| 4. treatment cost.mp. |
| 5. health service cost.mp. |
| 6. drug cost.mp. |
| 7. surgery cost.mp. |
| 8. chemotherapy cost.mp. |
| 9. radiotherapy cost.mp. |
| 10. endocrine cost.mp. |
| 11. targeted therapy cost.mp. |
| 12. (Cost or treatment cost or health service cost or drug cost or surgery cost or chemotherapy cost or radiotherapy cost or endocrine cost or targeted therapy cost).mp. [mp=title, abstract, original title, name of substance word, subject heading word, floating sub-heading word, keyword heading word, organism supplementary concept word, protocol supplementary concept word, rare disease supplementary concept word, unique identifier, synonyms] |
| 13. 3 or 4 or 5 or 6 or 7 or 8 or 9 or 10 or 11 |
| 14. 1 or 2 or 3 |
| 15. Melanoma/ or melanoma.mp. |
| 16. skin cancer.mp. or Skin Neoplasms/ |
| 17. basal cell carcinoma.mp. or Carcinoma, Basal Cell/ |
| 18. squamous cell carcinoma.mp. or Carcinoma, Squamous Cell/ |
| 19. 15 or 16 or 17 or 18 |
| 20. 14 and 19 |
| 21. 20 and 2015:2020.(sa_year). |

**Table S7.2: paper reporting annual UK treatment costs for skin cancer**

| **Author (year)** | **Title** | **Data sources and price year** |
| --- | --- | --- |
| Morris et al. (2009) | Cost of skin cancer in England | HES data and NHS unit costs from 2002 |
| Vallejo-Torres et al. (2014) | Measuring current and future cost of skin cancer in England | HES data and NHS unit costs from 2002 – Top down and bottom up approaches used |
| Johnston et al. (2012) | Economic impact of healthcare resource utilisation patterns  among patients diagnosed with advanced melanoma in the  United Kingdom, Italy, and France: Results from a retrospective, longitudinal survey (MELODY study) | NHS reference costs from 2009 used for resources used by UK participants |
| Wilson et al. (2018) | A Modeling Study of the Cost-Effectiveness of a Risk-Stratified Surveillance Program for Melanoma in the United Kingdom | NHS units costs (price year 2015) attached to resource use from care pathways and data from recent trial |
| Krensal et al. (2019a) | Cost-of-illness of melanoma in Europe - a systematic review of the published literature | Draws on Johnston et al. (2012), Vallejo-Torres et al. (2014) and Morris et al. (2009) |
| Krensal et al. (2019b) | Cost-of-illness of melanoma in Europe - a modelling approach | Draws on Johnston et al. (2012), Vallejo-Torres et al. (2014) and Morris et al. (2009) |
| Krensal et al. (2019c) | Modelling first-year cost-of-illness of melanoma attributable to sunbed use in Europe | Draws on Johnston et al. (2012), Vallejo-Torres et al. (2014) and Morris et al. (2009) |

From the three papers reporting empirical work to determine skin cancer costs, only one paper reported estimates for keratinocyte cancers (KC). Vellejo-Torres et al. (6) used top-down and bottom-up approaches to generate expected yearly costs of a KC. The mean of these two estimates (£1347) was used for the base case. Melanoma treatment costs reported by stage in Wilson et al. (4) were used in a weighted average for thin (£1338.07) and melanoma >1mms (£3182.02) using proportions of individuals in each cancer stage from Schoffer et al. (8). Each component cost was inflated to 2019 prices using the following formula: y = 5.8479x + 232.21 derived from 2004 to 2016 health sector cost rises (<https://nhsprocurement.org.uk/health-sector-cost-index-update>)

No costs were applied to lesion-free no-melanoma state, post-melanoma states^[[1]](#footnote-1)^ and for deaths unrelated to melanoma. Cost of death from melanoma was taken from final year of life costs from Wilson et al. (4)

**Table S6.3: component costs for melanoma treatments (from Wilson et al.)**

|  |  | ***deterministic (no inflation)*** | ***distribution*** | ***mean*** | ***Standard error*** |
| --- | --- | --- | --- | --- | --- |
| GP consultation (per min) |  | 3.8 |  |  |  |
| GP consultation time (min) |  | 22.1 | normal | 22.1 | 3 |
| GP consultation | £92.26 | 83.98 |  |  |  |
|  |  |  |  | *alpha* | *beta* |
| Initial referral | £121.94 | 111 | gamma | 20.408 | 5.439 |
| Biopsy excision | £157.10 | 143 | gamma | 10.443 | 13.694 |
| Definitive surgery | £171.38 | 156 | gamma | 3.762 | 41.468 |
| CXR | £32.96 | 30 | gamma | 12.43 | 2.414 |
| CT scan | £145.02 | 132 | gamma | 13.616 | 9.695 |
| Liver function test | £3.30 | 3 | gamma | 4.041 | 0.742 |
| FBC | £3.30 | 3 | gamma | 4.041 | 0.742 |
| Sentinel node biopsy | £31.86 | 29 | gamma | 1.165 | 24.887 |
| Radical lymph node dissection | £1,088.72 | 991 | gamma | 1.808 | 547.925 |
| Surgical removal of localized metastases | £796.49 | 725 | gamma | 1.256 | 577.101 |
| Radiotherapy (planning) | £807.47 | 735 | gamma | 8.89 | 82.673 |
| Radiotherapy (per fraction) | £145.02 | 132 | gamma | 17.014 | 7.758 |
| Chemotherapy (dacarbazine, procurement) | £306.51 | 279 | gamma | 1.33 | 209.827 |
| Chemotherapy (dacarbazine, delivery first attendance) | £204.34 | 186 | gamma | 6.934 | 26.823 |
| Chemotherapy (dacarbazine, delivery subsequent) | £224.12 | 204 | gamma | 3.239 | 62.988 |
| Dermatology follow-up | £106.56 | 97 | gamma | 12.183 | 7.962 |

*References*

1. Krensel M, Schäfer I, Augustin M. Cost-of-illness of melanoma in Europe - a modelling approach. J Eur Acad Dermatol Venereol. 2019 Mar;33 Suppl 2:34–45.

2. Krensel M, Schäfer I, Augustin M. Modelling first-year cost-of-illness of melanoma attributable to sunbed use in Europe. J Eur Acad Dermatol Venereol. 2019 Mar;33 Suppl 2:46–56.

3. Krensel M, Schäfer I, Augustin M. Cost-of-illness of melanoma in Europe - a systematic review of the published literature. J Eur Acad Dermatol Venereol. 2019 Mar;33(3):504–10.

4. Wilson ECF, Usher-Smith JA, Emery J, Corrie P, Walter FM. A Modeling Study of the Cost-Effectiveness of a Risk-Stratified Surveillance Program for Melanoma in the United Kingdom. Value Health. 2018;21(6):658–68.

5. Johnston K, Levy AR, Lorigan P, Maio M, Lebbe C, Middleton M, et al. Economic impact of healthcare resource utilisation patterns among patients diagnosed with advanced melanoma in the United Kingdom, Italy, and France: results from a retrospective, longitudinal survey (MELODY study). Eur J Cancer. 2012 Sep;48(14):2175–82.

6. Vallejo-Torres L, Morris S, Kinge JM, Poirier V, Verne J. Measuring current and future cost of skin cancer in England. J Public Health (Oxf). 2014 Mar;36(1):140–8.

| 7. Morris S, Cox B, Bosanquet N. Cost of skin cancer in England. Eur J Health Econ. 2009 Jul;10(3):267–73. |
| --- |

8. Schoffer O, Schülein S, Arand G, *et al.* Tumour stage distribution and survival of malignant melanoma in Germany 2002–2011. *BMC Cancer* 2016;**16**. doi:[10.1186/s12885-016-2963-0](https://doi.org/10.1186/s12885-016-2963-0)

**Appendix 8:** Utility multiplier decrements

*Aim:* to identify and apply cancer-related utility multiplier decrements to age-based health related quality of life in each cohort

*Method:* Using utility values reported in Wilson et al. (1) a weighted average utility for thin and melanoma >1mm was calculated based on proportion of individuals in each cancer stage from Schoffer et al. (2) (see table S7.1). Based on expert opinion the disutility associated with a keratinocyte cancer was assumed equal to that experienced for a thin melanoma.

*Results:* A utility decrement of 0.94 for thin melanoma, 0.94 for KC and 0.84 for melanoma >1mm (see table S8.1). These multiplier decrements were applied to age-specific population norm values in melanoma >1mm, thin melanoma and KC states (i.e. 0.94*population norm, 0.84*population norm & 0.84* population norm).

**Table S8.1: utility multiplier decrements**

|  | **Stage** | **Proportion in each stage from Schoffer et al. 2016)** | **Utility values from Wilson et al. (2018)** | **Weighted average** |
| --- | --- | --- | --- | --- |
| Thin melanoma | 0 | 23.18% | 0.93 | 0.94 |
|  | 1a | 26.00% | 0.93 |  |
|  | 1b | 26.00% | 0.93 |  |
| Melanoma >1mm | 2a | 4.96% | 0.87 | 0.84 |
|  | 2b | 4.96% | 0.87 |  |
|  | 2c | 4.96% | 0.87 |  |
|  | 3a | 2.40% | 0.89 |  |
|  | 3b | 2.40% | 0.89 |  |
|  | 3c | 2.40% | 0.89 |  |
|  | 4 | 2.75% | 0.52 |  |
| KC | NA | | | 0.94* |

*assumed equivalent to thin melanoma

*References*

1. Wilson ECF, Usher-Smith JA, Emery J, Corrie P, Walter FM. A Modeling Study of the Cost-Effectiveness of a Risk-Stratified Surveillance Program for Melanoma in the United Kingdom. Value Health. 2018;21(6):658–68.

2. Schoffer O, Schülein S, Arand G, *et al.* Tumour stage distribution and survival of malignant melanoma in Germany 2002–2011. *BMC Cancer* 2016;**16**. doi:[10.1186/s12885-016-2963-0](https://doi.org/10.1186/s12885-016-2963-0)

**Appendix 9:** Probabilistic sensitivity analysis

*Aim:* to characterise and quantify uncertainty in parameter inputs in order to calculate the likelihood of the intervention’s cost-effectiveness

*Method:* In a probabilistic sensitivity analysis, all relevant input parameters are varied simultaneously by randomly drawing from a range and distribution of plausible values. Ranges and distributions for included variables are described in Table 2 of the main article and in this appendix as indicated in Table 2. Simultaneous variation of input parameters in 5000 iterations of the model was used to generate estimates of uncertainty.

A mean incremental cost-effectiveness ratio (ICER) was calculated based on the 5000 iterations of the model. ICERs from each iteration were plotted on a cost-effectiveness plane and compared to the lower bound of the NICE cost-effectiveness threshold. The likelihood of the intervention’s cost-effectiveness was quantified by determining the proportion of estimates that fell below the NICE-recommended threshold of acceptability (£20000 to £30000). A bootstrapped 95% confidence interval around the mean ICER was generated.

Cost-effectiveness acceptability curves (CEACs) were produced to illustrate probability of cost-effectiveness based on PSA results. In a CEAC the proportion of ICERs from the PSA for both the intervention and current practice (y-axis) are plotted against a range of cost-effectiveness thresholds (x-axis).

A mean incremental net benefit was calculated based on the 5000 iterations of the model. A bootstrapped 95% confidence interval around the mean INB was generated.

*Input parameters for PSA:* As indicated in Table 2 of the main article, further detail is now provided around how specific parameters were varied in the PSA.

*Sunbed use prevalence:* An age-specific model for both sexes combined in England was developed based on estimates of starting prevalence and starting incidence, updated to 2020 and an assumed drop in incidence with age. A ratio of male to female use was then used as weightings to derive the sex-specific models. In total five parameters were used: 1) starting prevalence, 2) starting incidence, 3) drop in incidence with years of age, 4) two-yearly trend, and 5) female to male ratio of sunbed use. Upper and lower estimates of age-specific use in each model were derived by simultaneously setting each of the model parameters – except female to male ratio - to their upper and then lower quintiles. For the female to male ratio upper and lower bounds of a 95%CI was used (see Table S9.1). In the PSA each component of sunbed prevalence was simultaneously varied.

**Table S9.1: Central, lower and upper estimates used to derive sunbed prevalence curves**

|  | **Point estimate** | **Distribution** | **Parameter values** | **Lower quintile (95%CI)** | **Upper quintile (95%CI)** |
| --- | --- | --- | --- | --- | --- |
| Starting prevalence | 0.0491 | Beta | a. 1.25 b.24.2 | 0.0145 | 0.0779 |
| Starting incidence | 0.0170 | Normal | 0.0017 SE | 0.0156 | 0.0184 |
| Drop in incidence with years of age | 0.0800 | Normal | 0.008 SE | 0.0867 | 0.0733 |
| Biannual trend | 0.8644 | Normal | 0.08644 SE | 0.7917 | 0.9372 |
| Male: Female ratio | 2.1100 | Normal | 0.422 SE | (1.2829) | (2.9371) |

*Skin cancer utility multiplier decrements:* Each stage-specific utility weight from Wilson et al.’s study (1) was varied in the PSA before weighted averages for thin and melanoma >1mm were recalculated in each PSA iteration. See Table S9.2 for parameter values used.

**Table S9.2: utility values for melanoma (adapted from Wilson et al.*)**

|  | **Stage** | **Distribution** | **Mean** | **Standard error** |
| --- | --- | --- | --- | --- |
| Thin melanoma ^a^ | 0 | normal | 0.93 | 0.013 |
|  | 1a | normal | 0.93 | 0.013 |
|  | 1b | normal | 0.93 | 0.013 |
| Melanoma >1mm ^b^ | 2a | normal | 0.87 | 0.057 |
|  | 2b | normal | 0.87 | 0.057 |
|  | 2c | normal | 0.87 | 0.057 |
|  | 3a | normal | 0.89 | 0.046 |
|  | 3b | normal | 0.89 | 0.046 |
|  | 3c | normal | 0.89 | 0.046 |
|  | 4 | normal | 0.52 | 0.117 |

a. weighted average of values for stages 0 and 1

b. weighted average of values for stages 2,3 and 4

*Treatment costs:* Each component treatment cost was varied in the PSA to calculate new weighted average costs for thin melanoma and melanoma >1mm based on stage-specific costs reported in Wilson et al.’s study (1). See Table S9.3 for parameter values used to vary component treatment costs.

**Table S9.3 parameter values for component unit costs from Wilson et al. (2018) (1).**

|  |  | ***deterministic (no inflation)*** | ***distribution*** | ***mean*** | ***Standard error*** |
| --- | --- | --- | --- | --- | --- |
| GP consultation (per min) |  | 3.8 |  |  |  |
| GP consultation time (min) |  | 22.1 | normal | 22.1 | 3 |
| GP consultation | £92.26 | 83.98 |  |  |  |
|  |  |  |  | *alpha* | *beta* |
| Initial referral | £121.94 | 111 | gamma | 20.408 | 5.439 |
| Biopsy excision | £157.10 | 143 | gamma | 10.443 | 13.694 |
| Definitive surgery | £171.38 | 156 | gamma | 3.762 | 41.468 |
| CXR | £32.96 | 30 | gamma | 12.43 | 2.414 |
| CT scan | £145.02 | 132 | gamma | 13.616 | 9.695 |
| Liver function test | £3.30 | 3 | gamma | 4.041 | 0.742 |
| FBC | £3.30 | 3 | gamma | 4.041 | 0.742 |
| Sentinel node biopsy | £31.86 | 29 | gamma | 1.165 | 24.887 |
| Radical lymph node dissection | £1,088.72 | 991 | gamma | 1.808 | 547.925 |
| Surgical removal of localized metastases | £796.49 | 725 | gamma | 1.256 | 577.101 |
| Radiotherapy (planning) | £807.47 | 735 | gamma | 8.89 | 82.673 |
| Radiotherapy (per fraction) | £145.02 | 132 | gamma | 17.014 | 7.758 |
| Chemotherapy (dacarbazine, procurement) | £306.51 | 279 | gamma | 1.33 | 209.827 |
| Chemotherapy (dacarbazine, delivery first attendance) | £204.34 | 186 | gamma | 6.934 | 26.823 |
| Chemotherapy (dacarbazine, delivery subsequent) | £224.12 | 204 | gamma | 3.239 | 62.988 |
| Dermatology follow-up | £106.56 | 97 | gamma | 12.183 | 7.962 |

*Results:* The mean ICER derived from 5000 PSA iterations of the England model was negative. A majority of ICER estimates fall in the southeast quadrant of the plane indicating that the intervention would prove more effective and less expensive. Estimates in the northeast quadrant suggest it would be more expensive and more effective. A minority of estimates fall in the northwest quadrant indicating less effect at a higher cost (see Figure S9.1)

**Figure S9.1: cost-effectiveness plane**


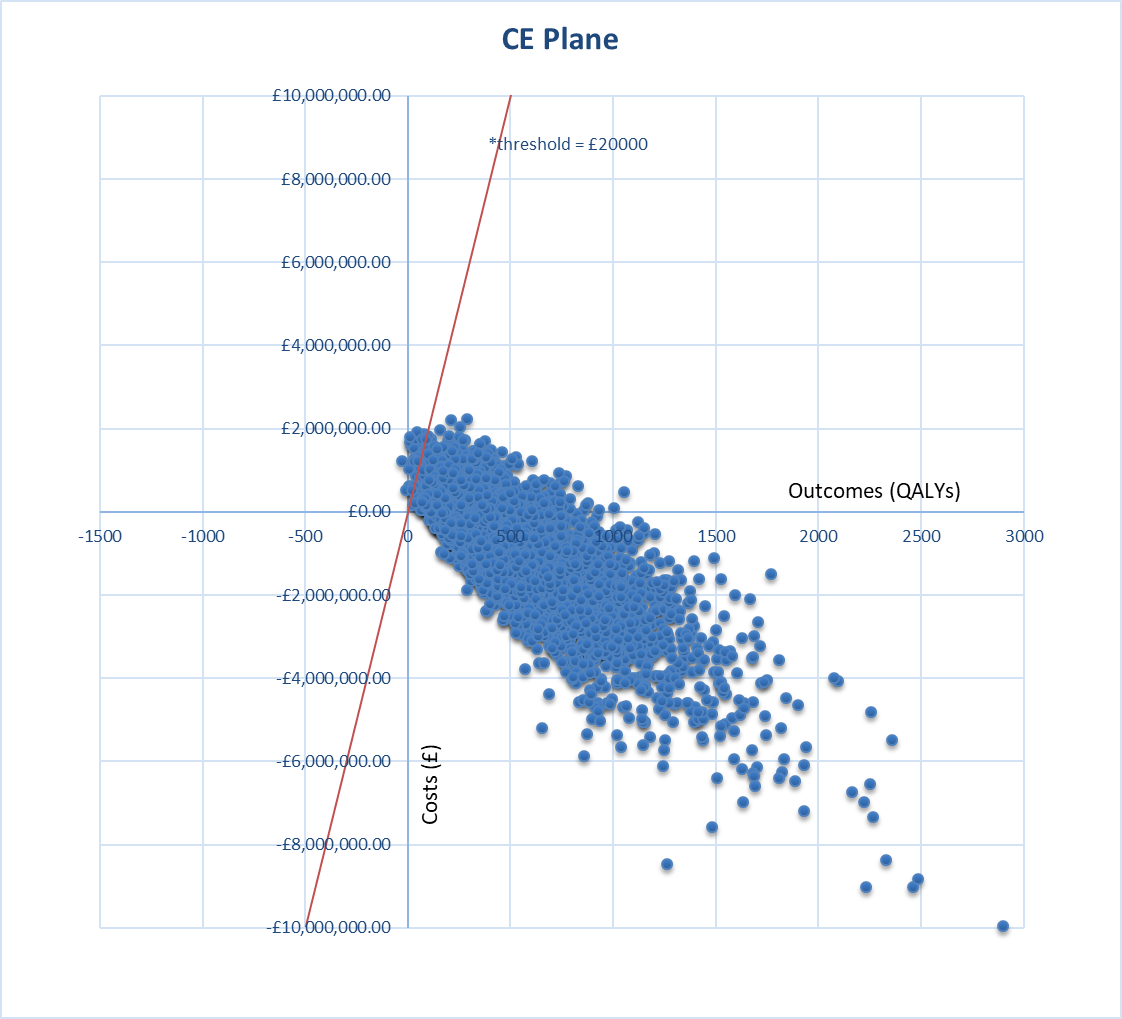


The proportion of PSA results falling below a range of cost-effectiveness thresholds for both the control and intervention arms are used to generate the cost-effectiveness acceptability curve in Figure S9.2. This graph shows that at a threshold of £20000 per QALY there is a 99.95% probability of the intervention being cost-effective in the England context. This likelihood increases to 99.97% at the outer £30000 bound of the NICE threshold (see Table S9.4)

**Figure S9.2: cost-effectiveness acceptability curve**


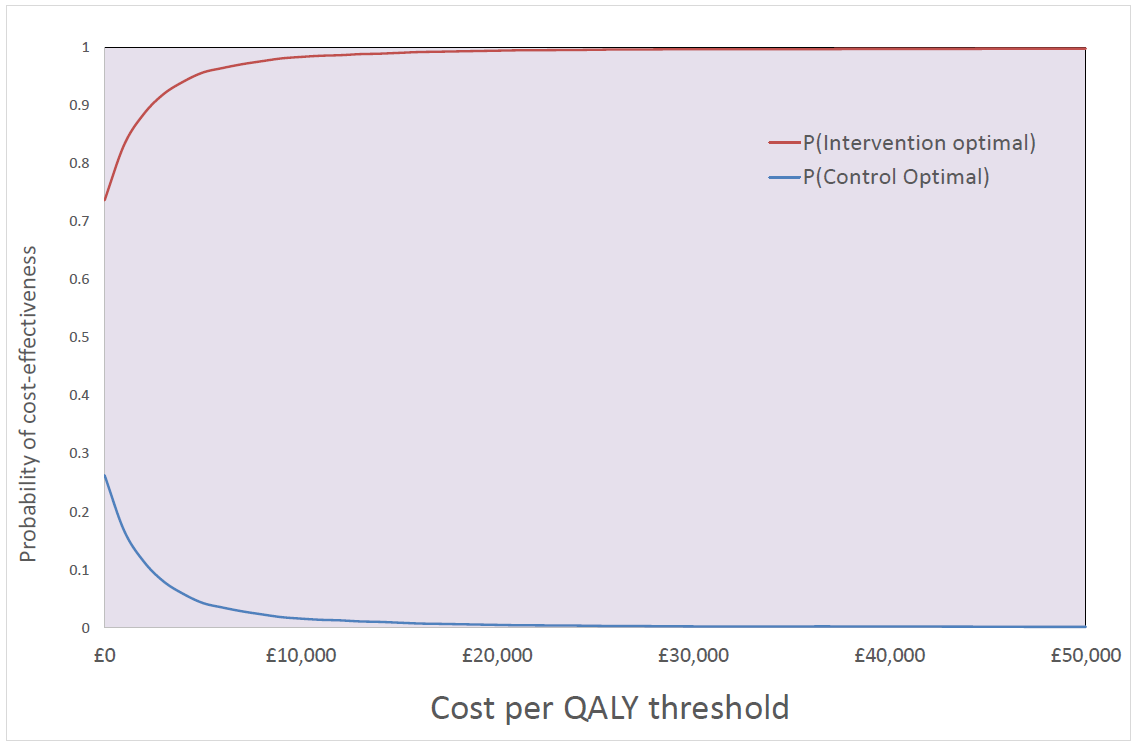


**Table S9.4: deterministic and probabilistic results**

|  | **Current Situation** | **Intervention** |
| --- | --- | --- |
| Deterministic Costs | £41,618,865.13 | £40,961,007.40 |
| Deterministic QALYS | 13619953 | 13620450 |
| Probabilistic Costs | £41,790,480.41 | £40,922,074.83 |
| Probabilistic QALYS | 13619936 | 13620470 |
| Deterministic ICER | NA | -£1324 (intervention cost-saving with QALY gain) |
| Probabilistic ICER (95%CI*) | NA | -£1626  (-£4410 to £7691) (intervention cost-saving with QALY gain) |
| Probability of cost-effectiveness at £20000 threshold | 0.005 | 0.995 |
| Probability of cost-effectiveness at £30000 threshold | 0.003 | 0.997 |
| Deterministic Incremental net benefit | NA | £10,599,040 |
| Probabilistic Incremental net benefit (95%CI*) | NA | £11,550,917  (£1,364,051 to £30,138,071) |
| *bootstrapped 95%CI |  |  |

1. Wilson ECF, Usher-Smith JA, Emery J, Corrie P, Walter FM. A Modeling Study of the Cost-Effectiveness of a Risk-Stratified Surveillance Program for Melanoma in the United Kingdom. Value Health. 2018;21(6):658–68.

**Appendix 10:** One-way sensitivity analysis inputs

*Aim:* the aim of the deterministic one-way sensitivity analysis was to identify key drivers of cost-effectiveness

*Method:* the state transition Markov model was re-run whilst systematically selecting extreme values for key variables. Extreme values were based on estimates of variance in the literature and use of assumptions where necessary. Extreme values are provided in Table S10.1 and additional detail is provided in the text below.

Table S10.1: inputs for one-way sensitivity analysis and range used

| **Model input parameter^a^** |  |  |
| --- | --- | --- |
|  | **worst-case estimate** | **best-case estimate** |
| Female to male sunbed use ratio | 1.3 | 2.9 |
| Sunbed use: current situation | Low^c^ | High^d^ |
| Sunbed use: intervention | 0.03 | 0.01 |
| Proportion of melanoma >1mm | 0.25 | 0.46 |
| Relative risk of melanoma | 1.36 | 1.85 |
| Relative risk of keratinocyte cancer | 1.21 | 2.08 |
| First year mortality risk: melanoma >1mm | 0.0446 | 0.0664 |
| Disutility multiplier: keratinocyte cancer | 0.90 | 0.96 |
| Disutility multiplier: thin melanoma | 0.90 | 0.96 |
| Disutility multiplier: melanoma >1mm | 0.77 | 0.91 |
| Treatment cost: keratinocyte cancer | £1083.66 | £1612.00 |
| Treatment cost: thin melanoma | £1075.81 | £1600.32 |
| Treatment cost: melanoma >1mm | £2558.36 | £3805.68 |
| Cost of death: melanoma | £3767.19 | £5603.89 |
| Campaign cost | £3,339,807 | £0 |

*Female to male ratio of sunbed use:* The lower and upper bounds of the 95% confidence interval around the point estimate ratio of female to male sunbed use (1) was used to generate low and high estimates for use in the deterministic sensitivity analysis.

*Sunbed use prevalence: current situation:* Firstly, an age-specific model for both sexes combined in England was developed based on estimates of starting prevalence and starting incidence, updated to 2020 and an assumed drop in incidence with age. A ratio of male to female use was then used as weightings to derive the sex-specific models. In total five parameters were used: 1) starting prevalence, 2) starting incidence, 3) drop in incidence with years of age, 4) biannual trend, and 5) male: female ratios of sunbed use.

Upper and lower estimates of age-specific use in each model were derived by simultaneously setting each of the model parameters to their upper and then lower quintiles. At the lower extreme an ever use prevalence of 0.0098 for a 18 year-old woman would be seen compared to a prevalence of 0.0869 in the higher estimate

*Sunbed use prevalence (intervention):* An assumed post intervention low estimate of 1% prevalence and a high of 3% sunbed use prevalence was assumed based on assumptions from previous economic evaluation (2).

*Proportion of melanoma >1mm:* Extreme values for the proportion of melanoma >1mm were generated using a beta distribution (a. 28.3 b. 51.7) around the point estimation from Sacchetto et al. (3).

*Relative risk of skin cancer attributable to sunbed use:* The lower and upper bounds of the 95% confidence intervals around the point estimates relative risk of melanoma (4) and KC (5) from sunbed use was used to generate low and high estimates for use in the deterministic sensitivity analysis.

*Skin cancer mortality risk:* The risk of death from melanoma in the year following a diagnosis was varied by ±~20% to generate extreme values for the deterministic sensitivity analyses. This initial mortality risk also drives the subsequent declining risk of death from melanoma >1mm and lifetime risk of death from thin melanoma.

*Disutility multiplier decrements:* In the one-way sensitivity analysis, multiplier decrements were varied using the upper and lower bounds of a 95%CI based on a standard error of 10% of the mean value for each multiplier.

*Skin cancer treatment costs:* In the one way sensitivity analysis skin cancer treatment costs were varied using the upper and lower bounds of a 95%CI based on a standard error of 10% of the mean value of costs for melanoma >1mm, thin melanoma and KCs.

*Campaign costs:* In the one way sensitivity analysis campaign costs were varied using a £0 value for the lower bound representing a situation where the NHS did not bear any campaign cost and an upper bound of £3.34 million based on the highest annual spend on the Stoptober campaign in 2015 (£3.1 million inflated to 2019 prices).

*References*

1. Suppa M, Gandini S, Njimi H, *et al.* Prevalence and determinants of sunbed use in thirty European countries: data from the Euromelanoma skin cancer prevention campaign. *Journal of the European Academy of Dermatology and Venereology* 2019;**33**:13–27. doi:[10.1111/jdv.15311](https://doi.org/10.1111/jdv.15311)

*2.* Gordon LG, Rodriguez-Acevedo AJ, Køster B, *et al.* Association of Indoor Tanning Regulations With Health and Economic Outcomes in North America and Europe. *JAMA Dermatol* Published Online First: 19 February 2020. doi:[10.1001/jamadermatol.2020.0001](https://doi.org/10.1001/jamadermatol.2020.0001)

3. Sacchetto L, Zanetti R, Comber H, *et al.* Trends in incidence of thick, thin and in situ melanoma in Europe. *European Journal of Cancer* 2018;**92**:108–18. doi:[10.1016/j.ejca.2017.12.024](https://doi.org/10.1016/j.ejca.2017.12.024)

4. Boniol M, Autier P, Boyle P, *et al.* Cutaneous melanoma attributable to sunbed use: systematic review and meta-analysis. *BMJ* 2012;**345**:e4757.

5. Wehner MR, Shive ML, Chren M-M, *et al.* Indoor tanning and non-melanoma skin cancer: systematic review and meta-analysis. *BMJ* 2012;**345**:e5909. doi:[10.1136/bmj.e5909](https://doi.org/10.1136/bmj.e5909)

**Appendix 11:** Two-way sensitivity analysis

*Aim:* to explore the impact of a simultaneous change in cost and effect of treatment for melanoma >1mm

*Method:* the analysis was re-run with cost and effect parameters being systematically varied. Cost of treating melanoma >1mm was increased in 20% increments until they were inflated by 200% from the base case estimate. Treatment effect (reduced risk of dying from melanoma >1mm in the year following diagnosis) was improved in 5% increments until a 50% improvement was seen. The Incremental Net Benefit (INB) was calculated for each combination of cost and effect increases.

*Results:* The INB for each cost and effect combination is shown in table S11.1

**Table S11.1: results from the two-way sensitivity analysis (Incremental Net Benefit)**

|  |  |  | **Treatment effect (reduction in risk of death from melanoma >1mm in year 1)** | | | | | | | | | | | |
| --- | --- | --- | --- | --- | --- | --- | --- | --- | --- | --- | --- | --- | --- | --- |
|  |  |  | 5% | 10% | 15% | 20% | 25% | 30% | 35% | 40% | 45% | 50% |  |  |
|  |  |  | 0.052725 | 0.04995 | 0.047175 | 0.0444 | 0.041625 | 0.03885 | 0.036075 | 0.0333 | 0.030525 | 0.02775 |  |  |
| Treatment costs for melanoma >1mm | 200% | £9,546 | £10,773,988 | £10,378,183 | £9,977,062 | £9,570,539 | £9,158,529 | £8,740,945 | £8,317,697 | £7,888,696 | £7,453,850 | £7,013,066 |  |  |
|  | 180% | £8,910 | £10,717,426 | £10,321,610 | £9,920,478 | £9,513,944 | £9,101,923 | £8,684,327 | £8,261,069 | £7,832,056 | £7,397,199 | £6,956,402 |  |  |
|  | 160% | £8,273 | £10,660,863 | £10,265,037 | £9,863,894 | £9,457,349 | £9,045,317 | £8,627,710 | £8,204,440 | £7,775,416 | £7,340,547 | £6,899,738 |  |  |
|  | 140% | £7,637 | £10,604,301 | £10,208,464 | £9,807,310 | £9,400,754 | £8,988,711 | £8,571,093 | £8,147,811 | £7,718,776 | £7,283,895 | £6,843,074 |  |  |
|  | 120% | £7,000 | £10,547,738 | £10,151,891 | £9,750,726 | £9,344,159 | £8,932,105 | £8,514,476 | £8,091,183 | £7,662,136 | £7,227,243 | £6,786,411 |  |  |
|  | 100% | £6,364 | £10,491,176 | £10,095,318 | £9,694,142 | £9,287,564 | £8,875,499 | £8,457,858 | £8,034,554 | £7,605,495 | £7,170,591 | £6,729,747 |  |  |
|  | 80% | £5,728 | £10,434,613 | £10,038,744 | £9,637,558 | £9,230,970 | £8,818,893 | £8,401,241 | £7,977,925 | £7,548,855 | £7,113,939 | £6,673,083 |  |  |
|  | 60% | £5,091 | £10,378,051 | £9,982,171 | £9,580,974 | £9,174,375 | £8,762,287 | £8,344,624 | £7,921,297 | £7,492,215 | £7,057,287 | £6,616,419 |  |  |
|  | 40% | £4,455 | £10,321,488 | £9,925,598 | £9,524,390 | £9,117,780 | £8,705,681 | £8,288,007 | £7,864,668 | £7,435,575 | £7,000,635 | £6,559,755 |  |  |
|  | 20% | £3,818 | £10,264,926 | £9,869,025 | £9,467,806 | £9,061,185 | £8,649,075 | £8,231,390 | £7,808,039 | £7,378,934 | £6,943,983 | £6,503,091 |  |  |

**Appendix 12:** Scenario analysis applying disutility for denial of sunbed use

*Aim:* to determine the extent of disutility (HRQL decrement) needed to be experienced by those denied use of sunbeds who would be expected to be regular users to influence decision on the intervention’s cost-effectiveness.

*Method:* utility values were reduced in 0.5% decrements and the state transition Markov model re-rerun each time. These utility decrements were applied to the proportion of the cohort not using sunbeds post-ban but who would likely be regular users in the current situation. The utility decrement was applied in the year in which first use would have occurred for this proportion of the cohort. The Incremental Net Benefit (INB) for each iteration of the model is shown in Table S12.1

**Table 12.1: effect of disutility from denial of use on INB**

|  | Control | | Intervention | | |
| --- | --- | --- | --- | --- | --- |
|  | Costs | QALYs | Costs | QALYs | INB |
| HRQL depleted* by 0.5% | £41,618,865 | 13619953 | £40,961,007 | 13620414 | £9,860,814 |
| HRQL depleted* by 1.0% | £41,618,865 | 13619953 | £40,961,007 | 13620377 | £9,122,589 |
| HRQL depleted* by 1.5% | £41,618,865 | 13619953 | £40,961,007 | 13620340 | £8,384,363 |
| HRQL depleted* by 2.0% | £41,618,865 | 13619953 | £40,961,007 | 13620303 | £7,646,138 |
| HRQL depleted* by 2.5% | £41,618,865 | 13619953 | £40,961,007 | 13620266 | £6,907,912 |
| HRQL depleted* by 3.0% | £41,618,865 | 13619953 | £40,961,007 | 13620229 | £6,169,686 |
| HRQL depleted* by 3.5% | £41,618,865 | 13619953 | £40,961,007 | 13620192 | £5,431,461 |
| HRQL depleted* by 4.0% | £41,618,865 | 13619953 | £40,961,007 | 13620155 | £4,693,235 |
| HRQL depleted* by 4.5% | £41,618,865 | 13619953 | £40,961,007 | 13620118 | £3,955,010 |
| HRQL depleted* by 5.0% | £41,618,865 | 13619953 | £40,961,007 | 13620081 | £3,216,784 |
| HRQL depleted* by 5.5% | £41,618,865 | 13619953 | £40,961,007 | 13620044 | £2,478,559 |
| HRQL depleted* by 6.0% | £41,618,865 | 13619953 | £40,961,007 | 13620008 | £1,740,333 |
| HRQL depleted* by 6.5% | £41,618,865 | 13619953 | £40,961,007 | 13619971 | £1,002,107 |
| HRQL depleted* by 7.0% | £41,618,865 | 13619953 | £40,961,007 | 13619934 | £263,882 |
| HRQL depleted* by 7.5% | £41,618,865 | 13619953 | £40,961,007 | 13619897 | -£474,344 |
| HRQL depleted* by 8.0% | £41,618,865 | 13619953 | £40,961,007 | 13619860 | -£1,212,569 |
| HRQL depleted* by 8.5% | £41,618,865 | 13619953 | £40,961,007 | 13619823 | -£1,950,795 |
| HRQL depleted* by 9.0% | £41,618,865 | 13619953 | £40,961,007 | 13619786 | -£2,689,021 |
| HRQL depleted* by 9.5% | £41,618,865 | 13619953 | £40,961,007 | 13619749 | -£3,427,246 |
| HRQL depleted* by 10.0% | £41,618,865 | 13619953 | £40,961,007 | 13619712 | -£4,165,472 |

*in year when 1st use would have occurred

1. Cost of all follow-ups were applied in year of diagnosis [↑](#footnote-ref-1)
